# Recent Advances in Metaphotonic Biosensors

**DOI:** 10.3390/bios13060631

**Published:** 2023-06-07

**Authors:** Dang Du Nguyen, Seho Lee, Inki Kim

**Affiliations:** 1 Department of Biophysics, Institute of Quantum Biophysics, Sungkyunkwan University, Suwon 16419, Republic of Korea; 2 Department of Intelligent Precision Healthcare Convergence, Sungkyunkwan University, Suwon 16419, Republic of Korea

**Keywords:** metaphotonic surface, biosensing, refractometric sensing, surface-enhanced fluorescence sensing, surface-enhanced vibrational spectroscopy, chiral sensing

## Abstract

Metaphotonic devices, which enable light manipulation at a subwavelength scale and enhance light–matter interactions, have been emerging as a critical pillar in biosensing. Researchers have been attracted to metaphotonic biosensors, as they solve the limitations of the existing bioanalytical techniques, including the sensitivity, selectivity, and detection limit. Here, we briefly introduce types of metasurfaces utilized in various metaphotonic biomolecular sensing domains such as refractometry, surface-enhanced fluorescence, vibrational spectroscopy, and chiral sensing. Further, we list the prevalent working mechanisms of those metaphotonic bio-detection schemes. Furthermore, we summarize the recent progress in chip integration for metaphotonic biosensing to enable innovative point-of-care devices in healthcare. Finally, we discuss the impediments in metaphotonic biosensing, such as its cost effectiveness and treatment for intricate biospecimens, and present a prospect for potential directions for materializing these device strategies, significantly influencing clinical diagnostics in health and safety.

## 1. Introduction

The aim of metaphotonics is to develop new optical functionalities that are beyond the conventional optical behavior of naturally occurring materials via the creation of artificial subwavelength nanostructures [1,2]. These structures control the electromagnetic waves using electric and magnetic resonances as well as scattering, enabling innovative modulation of the useful properties of materials. It is possible to manipulate the efficient refractive index (RI) of metamaterials to be close to or below zero by adjusting the composition and arrangement of the constituent materials. Metaphotonics merges the notions of metamaterials and nanophotonics [3,4,5,6,7,8] to provide an exceptional platform for attaining anomalous electromagnetic responses. Conventionally, the effective regulation of light at subwavelength scales was obtained by plasmonic effects originating from metallic surfaces or interfaces [9]. Plasmonic structures create surface plasmons with intense near-field enhancement through a strong interaction with incident light. Over the past several years, significant advancements have been made in the highly efficient manipulation of light within the subwavelength dielectric structures. These developments have provided a fundamentally advanced perception of light–matter interactions at a subwavelength scale.

Optical metasurfaces composed of subwavelength artificial nanostructures, called meta-atoms, can manipulate the light–matter interactions with intended optical and photonic functionalities [10,11,12,13,14]. The periodical assembly of meta-atoms is crucial in defining the features of metaphotonic devices [15,16,17]. There is a strong desire for noble metal nanoparticles that demonstrate high sensitivities to the refractive index in the context of plasmon spectroscopy, primarily due to their significant impact on the detection sensitivity. The sensitivity of the spectral peak position to changes in the refractive index exhibits an inclination to increase with both the size of the nanoparticle and the resonance wavelength [18]. The refractive index sensitivity could be enhanced by modifying the shape of the nanoparticle and diverse configurations, such as nanorods, nanorings, and nanocubes [19]. For example, Singh et al. presented the excitation of guided and longitudinal plasmonic modes in the nanorods sensing layer, enabling the sensor to exhibit improved sensitivity and a narrow figure of merit performance [20]. The structural parameters of the nanorods can be controlled in the range of rod lengths ranging from 20 to 700 nm and rod diameters of 10–50 nm, thus obtaining a nanorod area density of around 10^10^–10^11^ cm^−2^. The utilization of nanorods also resulted in a larger surface area available for biomolecular sensing, leading to a substantial improvement in detection sensitivity. The sensor has been successfully utilized for the detection of streptavidin-biotin binding, yielding a detection limit of 300 nM. Moreover, an asymmetric split-ring array was employed to enhance the performance of metasurface-based sensors [21]. It stimulated low-loss quadrupole and Fano resonances, characterized by remarkably narrow linewidths, which empower the precise measurement of minute spectral shifts resulting from even the slightest changes in the refractive index of the surrounding media. Analyzing the size and shape parameters of metasurfaces in metaphotonic biosensing is crucial for optimizing their sensing performance, tailoring their interaction with analyte molecules, and achieving the desired sensitivity, selectivity, and detection limits. Recently, plasmonic metasurfaces (PMs) have been used in biosensing applications due to their distinct photonic capabilities [22,23,24,25,26]. The binding or adsorption of molecules can change the resonant frequency of a surface plasmon because it depends on the interfacial medium and properties, such as the local RI [27,28,29]. Therefore, it is possible to identify this adjusted surface plasmon resonance as an alteration in the absorption wavelength or the angle of minimal reflectivity. PMs can be functionalized to precisely capture target molecules, increasing their sensing capability. Thus, a broad range of functionalization techniques was developed to alter metals, such as gold, silver, and aluminum [30,31,32,33], that are frequently employed in the manufacture of PMs. These approaches typically incorporate either fixed gaps between nanoparticles to generate hot spots or gaps between surface chemical moieties for selectively attaching to a target analyte. Aluminum’s plasmonic performance is typically weak due to its lower electron density compared to noble metals. The lower electron density limits the magnitude of plasmonic effects, leading to reduced sensitivity and detection capabilities. However, by employing an array of aluminum-based nanodisks-in-cavities, a metasurface structure can attain a remarkable 1000-fold enhancement in fluorescence within the visible wavelength range [34]. The designed structure, which produced hybrid multipolar lossless plasmonic modes, facilitated robust electromagnetic field confinement. This metasurface sensor proved highly effective in detecting insulin, vascular endothelial, and thrombin biomarkers, even at low concentrations of 1 fmol in a 10 μL droplet. A recent study introduced a metasurface comprising multilayers of titanium, silver, and gold on a digital versatile disk surface [35]. The presence of a uniform grating periodicity on the surface significantly contributed to the facilitation of efficient light excitation and the coupling of surface plasmon resonance. This metasurface provides notable benefits such as cost-effectiveness, a large detection area, and the capability for multimodal sensing.

The emergence of resonant metaphotonics is revolutionizing for subwavelength optics [36]. Resonant dielectric structures offer several advantages over plasmonic analogues, including low-loss and increased magnetic field enhancement, imparting significant effects owing to strong coupling and interference in certain optical resonance modes [7]. Dielectric metasurfaces offer substantial benefits compared to plasmonic counterparts, providing an effective means of controlling optical properties, such as the amplitude, phase, and polarization [37]. Thus, dielectric metasurfaces present new possibilities for metaphotonic biosensing because of their remarkable optical characteristics. For instance, dielectric media are excellent candidates for the creation of highly sensitive refractometric sensors because of the exceptionally high transmission and RI [38]. The potential research areas of metaphotonics with plasmonic and dielectric effects include various advanced metaphotonic biological sensing techniques.

Diagnostic biosensors can continuously acquire biomedical data to aid in clinical judgment and health surveillance [39,40,41]. Traditional biosensors, such as labeled immunoassays and polymerase chain reactions (PCRs), are necessary in laboratories where there are many specimens and where a quick reaction is not required [42]. Despite being sensitive and accessible for many types of analytes, labeled immunoassays call for large and expensive benchtop equipment with lengthy multistep detection processes. Therefore, cost-effective, simple-to-use, and label-free optical biosensors will revolutionize the detection of diseases and enable their widespread application in almost any setting [43,44]. The application of integrated metaphotonic biosensors has become prevalent in recent years because they are inexpensive, highly sensitive, simple to read, and capable of ensuring superior surface biofunctionalization. Moreover, they are real-time detectors.

**Figure 1:**
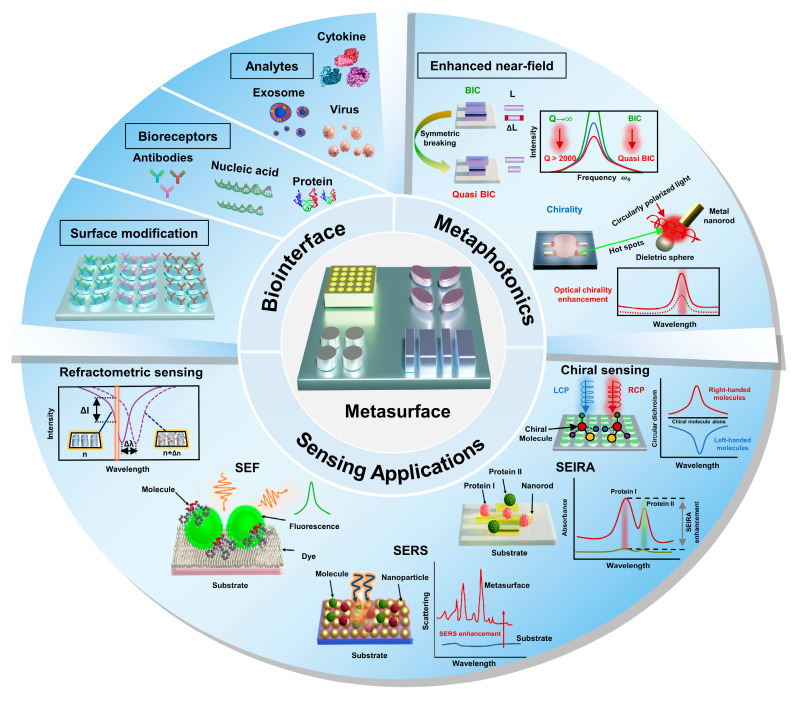
Diverse types of metasurfaces for metaphotonic biosensors. (Biointerface) Using a specialized bio-capture probe mounted on the metasurface, target analytes from various sources can be selectively caught, enabling sensitive and nondestructive detection. (Metaphotonics) Near-fields enhancement of quasibound states in the continuum (quasi-BIC) and chirality for ultrasensing performance. (Sensing applications) Major detection schemes include refractometric sensing, surface-enhanced fluorescence (SEF) sensing, surface-enhanced Raman spectroscopy (SERS), surface-enhanced infrared absorption (SEIRA) spectroscopy, and chiral sensing.

Metaphotonic biosensors enhance the light–matter interactions at nanometer scales to detect minuscule objects. They satisfy the requirement for building effective and dependable sensing systems to address the difficulties [45] regarding instantaneous biological tests [46] and point-of-care (POC) examination [47] in personalized healthcare. Ultrahigh sensitivity, selectivity, and a low detection limit are some of the main characteristics that can help the modern medical devices function well [48]. Surface functionalization and tunable configuration techniques are vital to every optical sensor because they improve selectivity and sensitivity. Generally, a metaphotonic biosensor is functionalized with specific bio-capture probes, such as proteins, nucleic acids, and antibodies, to detect a target analyte (Figure 1). This increases the selectivity and sensitivity of transduction. The transduction process produces an optical signal that is measured and then used in signal processing.

The phenomenon of electric and magnetic Mie-type resonances can be observed in high-index dielectric resonators, which possess the ability to be precisely tailored by manipulating the geometry of meta-atoms. The concept of bound states in the continuum (BICs) can be utilized to localize electromagnetic energy within subwavelength resonators. This is accomplished by achieving destructive interference between two leaky modes [49]. The utilization of metaphotonic nanostructures, comprising both metals and high-RI dielectrics, enables the significant enhancement of electromagnetic near-fields, which leads to an increase in light–matter interactions. Therefore, resonant metaphotonic systems have the essential characteristics in the creation of biosensing platforms. Biosensors have captured the attention of researchers due to their ability to address the constraints encountered in current bioanalytical techniques, such as the sensitivity, selectivity, and detection limit. However, a few applications of biosensors utilizing metaphotonic responses have been reported. For example, Wang et al. have focused on the underlying physical sensing mechanisms by plasmonic metasurfaces and highlight recent advancements in their application for cancer detection and COVID-19 detection [50]. Furthermore, Oh et al. provided a concise overview of nanophotonic sensing principles that rely on van der Waals (vdW) materials. Afterwards they proceed to discuss the subsequent technological hurdles, including surface chemistry, integration, and toxicity [51].

In this review, we present a summary of the fundamental principles, distinguishing features, and influential variables of various prominent resonance modes engendered by the interplay between electromagnetic waves and plasmons or dielectric meta-atoms, which enable refractometric, surface-enhanced fluorescence, surface-enhanced vibrational spectroscopic, and chiral sensing (Figure 1). Additionally, we highlight recent advancements in on-chip integrated metaphotonic-based biosensing technology for facilitating a novel category of healthcare devices in POC clinical diagnostics. Further, we discuss the difficulties associated with metaphotonic biosensing and forecast forthcoming pathways for advancing these devices.

**Figure 2:**
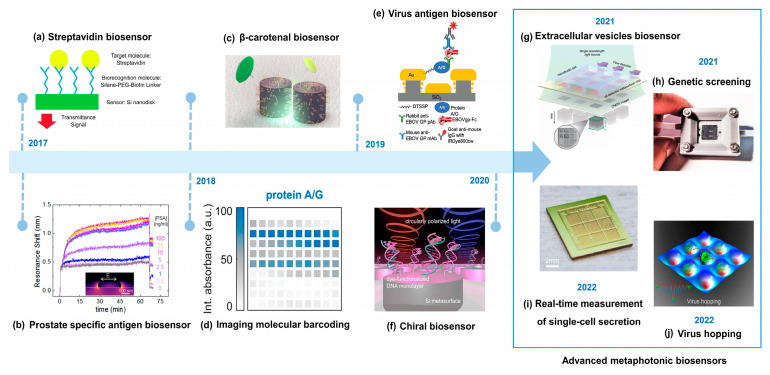
Roadmap highlighting the growth of metaphotonic biosensors. (**a**) Schematic of biosensing measurement with all-dielectric nanoresonators. (**a**) Reproduced with permission from [52]. Copyright © 2017 The Royal Society of Chemistry. (**b**) The detection of diverse concentrations of prostate-specific antigen (PSA) leads to dynamic changes in the resonance shifts of the silicon nanodisk arrays in real time and the different concentrations are listed such as 0 (gray), 0.5 (light magneta), 1 (blue), 2.5 (violet), 5 (royal), 10 (magneta), 25 (yellow), 100 (purple). (**b**) Reproduced with permission from [53]. Copyright © 2017 American Chemical Society. (**c**) Detection of the β-carotenal monolayer on silicon dimer nanostructures based on surface-enhanced spectroscopies. (**c**) Reproduced with permission from [54]. Copyright © 2018 American Chemical Society. (**d**) Identification of molecular barcodes associated with protein A/G through imaging-based techniques. Reproduced with permission from [55]. Copyright © 2018 The Authors. (**e**) Ebola virus antigen detection using a nanoantenna biosensor in a sandwich assay form. Reproduced with permission from [56]. © 2019 WILEY-VCH. (**f**) Schematic of chiral molecular monolayer detection using dielectric metasurfaces. Reproduced with permission from [57]. Copyright © 2020 American Chemical Society. (**g**) Illustration of a real-time in-flow imaging approach with all-dielectric sensors integrated with a microfluidic cell. Reproduced under CC BY Creative Commons Attribution 4.0 International License (https://creativecommons.org/licenses/by/4.0 accessed on 20 April 2023.) [58]. Copyright © 2021, The Author(s). Springer Nature. (**h**) Image of a metasurface chip securely enclosed within a fluid cell, designed for expeditious genetic screening. Reproduced under CC BY Creative Commons Attribution 4.0 International License (https://creativecommons.org/licenses/by/4.0 accessed on 20 April 2023.) [59]. Copyright 2021, arXiv. (**i**) Photo of the integrated biochip that encompasses nanoplasmonic gold nanohole arrays. Reproduced with permission from [60]. © 2022 Elsevier. (**j**) Movement of adenovirus as it transitions from the nearby hotspot to the central hotspot, characterized by a stronger laser intensity. Reproduced with permission from [61]. © 2022 Wiley-VCH.

Finally, we show the roadmap that illustrates the advancement of metaphotonic biosensors, depicting the chronological progression and key milestones achieved in the field in Figure 2.

## 2. Plasmonic and Dielectric Metasurfaces

According to classical electrodynamics, which is based on Maxwell’s equations, the light–matter interaction at the macroscopical level is well explained [62]. When light propagates from one medium to another, the speed of light changes at the boundary because of the optical properties of the medium, and light refraction and reflection occur [30]. These optical behaviors modify in the presence of metasurfaces. Because the metasurface is an incredibly thin and flat optical component, the optical properties of light passing across this interface vary. Additionally, the geometric configuration and order of meta-atoms affect the optical characteristics of the entire metasurface.

Metaphotonic nanostructures consisting of metal and/or high-RI dielectrics allow for the substantial enhancement of electromagnetic near-fields, which increase the light–matter interactions. Resonant metaphotonic systems possess the necessary characteristics to serve as optimal components for the development of label-free and non-invasive biosensing platforms. Subsequently, we will provide a concise overview of the underlying physical principles behind the concurrent enhancement of near-field spectra via using plasmonic and dielectric metasurfaces. Moreover, we will discuss the assembly of optical resonators forming different types of metasurfaces for biodetection applications.

### 2.1. Plasmonic Metasurfaces

#### 2.1.1. Surface Plasmon Resonance

**Figure 3:**
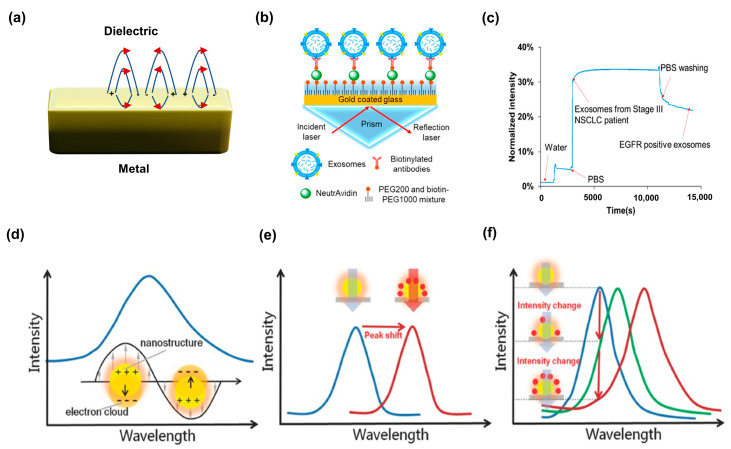
Plasmonic nanophotonic approaches. (**a**) Schematic of surface plasmon resonance (SPR) sustained at the interface of a metal and a dielectric material. (**b**) Sensing process of an SPR biosensor. (**c**) Exosomal EGFR detected in a serum sample from a Stage III lung cancer patient using an SPR biosensor with a real-time response curve. (**b**,**c**) Reproduced with permission from [67]. Copyright © 2018 American Chemical Society. (**d**) Interaction of the incident light with the electron clouds, resulting in a peak in the extinction spectra of nanostructures. (**e**) Peak shifts in the spectra might result from changes in RI caused by target molecules adhering to metallic surfaces (red). (**f**) Extinction intensity (green) at a given wavelength varies with the magnitude of the RI variations close to the LSPR sensing substrate and the spectra of nanostructures before attaching target molecules (blue). (**d**–**f**) Reproduced with permission from [70]. Copyright 2017, WILEY-VCH.

One of the major techniques for producing plasmonic excitation is metal–dielectric contact. It often propagates in a directed manner along metal–dielectric contacts [63]. Surface plasmon resonances (SPRs) pertain to the scrutiny of plasmons, which are elongated surface waves that traverse at the interface of a metallic surface with either air or glass (Figure 3a). When a target molecule binds to a sensor layer, SPR can be used to track the change in the RI of the layer [64]. To obtain SPR, the following resonance conditions must be satisfied [65]:

(1) $$\sin\theta_{res}\sqrt{\varepsilon_{p}}=\sqrt{\frac{\varepsilon_{d}\varepsilon_{m}}{\varepsilon_{d}+\varepsilon_{m}}},$$

where $\theta_{res}$ is the incident resonance angle, and $\varepsilon_{p}$, $\varepsilon_{d}$, and $\varepsilon_{m}$ are the dielectric constants of a substrate (e.g., optical fiber core, prism, etc.), dielectric layer (analyte medium), and plasmonic material (metals), respectively. The equation shows that the optical characteristics between a metal layer and a dielectric/sensing interface are responsive to changes in the position (angle or wavelength), depth, and phase of the examined SPR dip.

SPR research has increased dramatically, leading to the creation of numerous configurations and material mixtures that improve the performance of these sensors for POC applications. Recently, the nanoplasmonic exosome assay was conducted for diagnosing ovarian cancer [66]. However, the vast uses of nanoplasmonic biosensors, in contrast to conventional SPR biosensors, are constrained by the challenging and expensive nanostructure production. To identify proteins as potential biomarkers for diagnosing lung cancer, an intensity-modulated biosensor chip without nanostructures was developed [67]. The interaction between exosomal proteins and antibodies caused the change in the local RI, which affected the optical properties of surface plasmon modes. Consequently, these changes enabled the optical detection of exosomes (Figure 3b). A notable surge of reflection intensity was recorded, proving that exosomal EGFR could indeed be detected (Figure 3c).

#### 2.1.2. Localized Surface Plasmon Resonance

Localized Surface Plasmon Resonance (LSPR) is a different method for plasmonic biosensing, which is based on the confinement of light and the localized strengthening of the electromagnetic field in the immediate vicinity of the metallic surface [68]. The occurrence of the resonance at the intersection of incident light and electrons leads to the appearance of a peak in the vanishing spectra of the nanostructure (Figure 3d). The location of the peak is affected by the dielectric characteristics of the surroundings near the metallic surface, thereby demonstrating the sensitivity of the system [69]. A shift in the peak of the extinct spectra is caused by the adsorption of analytes, which results in alterations in the dielectric properties or RI (Figure 3e). The relationship between the shift in the peak wavelength ($∆\lambda_{max}$) and the alteration of RI (∆n) is explained by the following equation [69]:

(2) $$∆\lambda_{max}=m∆n[1-exp(-2d/l_{d})],$$

where *m* denotes the nanosubstrate’s sensitivity, ∆n is the alterations in RI occurring in the vicinity of a metallic surface, *d* is the adsorbate layer’s thickness, and $l_{d}$ corresponds to the duration required for the decay of the enhanced electromagnetic fields caused by the nanostructures. The widespread approach to determining an LSPR nanosubstrate’s RI sensitivity entails submerging it in liquids with different refractive indices and analyzing the resulting peak shifts. Signals for biological detection can be retrieved not only from the peak’s location but also from the intensity of extinction (Figure 3f) [70]. Depending on the concentration of the analyte nearby the metallic surface, the extinction intensity at a certain wavelength can alter appropriately.

### 2.2. Dielectric Metasurfaces

#### 2.2.1. Mie Resonances

**Figure 4:**
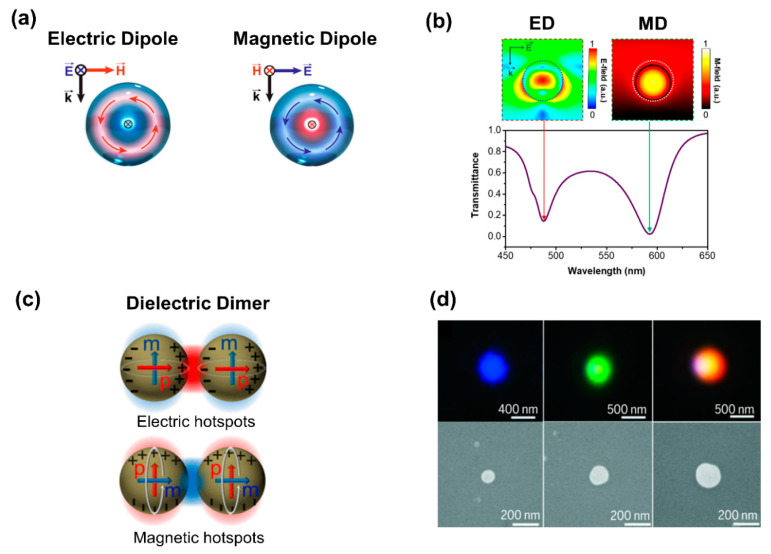
Mie resonances in high-index dielectric metasurfaces. (**a**) Diagrams illustrating the induced ED and MD in a spherical dielectric nanoparticle. The induced magnetic and electric fields inside the nanoparticle are indicated by the red and blue arrows, respectively. (**b**) Si spherical nanoparticle’s (150 nm diameter) transmission spectrum and resonance modes. (**a**,**b**) Reproduced with permission from [38]. Copyright © 2020 American Chemical Society. (**c**) Dielectric dimers possess electric as well as magnetic dipoles, which exhibit inter-coupling effects when subjected to polarizations that are perpendicular to each other. Reproduced with permission from [74]. Copyright © 2015 American Chemical Society (**d**) Spherical Si nanoparticles with varied diameters are shown: top image (a dark-field optical microscope) and bottom image (SEM). Reproduced with permission from [36]. Copyright © 2016, Science.

Dielectric nanostructures tailor optical fields via Mie-type resonance, based on Maxwell equations’ Mie solution. Typically, the nanostructure’s property dimension can be expressed as $\lambda /n_{eff}$, where $n_{eff}$ is the nanostructure’s effective RI and λ is its resonant wavelength. The utilization of dielectric materials with nanostructures possessing a high RI has surfaced as a potent means for the manipulation of light at the nanoscale [6,71,72]. This is particularly relevant for biosensing applications [38,73]. Since the characteristic size of the high-RI nanoparticles and the effective wavelength of the incident light are similar, the excitation of optical resonance can be accomplished by the oscillating displacement current. In particular, each sub-wavelength dielectric resonator may exhibit a robust response to light’s magnetic and electric fields. The plasmonic counterparts, on the other hand, have a weak magnetic field response. Therefore, the electric dipole (ED) and magnetic dipole (MD) can be observed in the spectrum of dielectric resonators in the Mie-type mode (Figure 4a) [38].

Silicon (Si) is a useful material for the investigation of Mie resonances, even though it is not totally loss-free and has a substantially lower visible absorption than metals. A spherical Si nanoparticle with a 150 nm diameter was simulated to produce its transmission spectrum and resonance modes (Figure 4b) [38]. Two dips can be seen in the transmission spectra, one at 490 nm for the electric mode and the other at 600 nm for the magnetic mode. The investigation of a nanodisk that is illuminated by a plane wave demonstrated the characteristic light scattering by nanoparticles.

The geometric configuration of dielectric resonators offers a means of tailoring the electric and magnetic multipole modes, thereby unveiling numerous nanophotonic designs that are highly versatile [6,7]. Numerical and experimental studies have demonstrated that the combination of both mode classes in a high-RI dielectric nanoparticle dimer, with a nanoscale gap, could result in a simultaneous two-order magnitude enhancement of both electric and magnetic field intensities (Figure 4c) [74]. At visible wavelengths, the electric and magnetic hotspots generated by the aforementioned phenomenon can significantly elevate the localized optical densities of states. As a result, these alter the rates at which molecules or emitters undergo magnetic transitions, which in turn improves the Purcell factor [75]. Because of these benefits, low-loss dielectric devices are highly appropriate for the recognition of biological species, without the concomitant generation and transfer of heat within the system [76].

Dark-field microscopy photos show various colors (Figure 4d) that are MD resonances of almost spherical Si nanoparticles [36]. The spherical nanoparticles exhibit a diverse range of vibrant hues. The red and green portions in the photos correspond to Si nanoparticles with different sizes. Dielectric metasurfaces’ resonant wavelengths can be precisely tuned over the visible [77] and infrared (IR) [78] spectra by altering the dimensions of constituent meta-atoms and the materials employed, as dictated by different sensing techniques. This advantageous characteristic facilitates the creation of a diverse array of sensing metasurfaces.

#### 2.2.2. Bound States in the Continuum

In the metaphotonic sensing realm, simple geometric dielectric resonators, such as spheres or disks, are still substantially constrained by electromagnetic field confinement within their internal structures. Consequently, there is limited overlap with the targeted analytes, and due to radiative damping, the resonance linewidths are relatively broad. Recently, to overcome these limitations, photonic bound states in the continuum (BICs) have been proposed and realized in all-dielectric nanostructured metasurfaces [79]. These BICs contrive resonant coupling with the far-field, thereby minimizing radiative loss.

**Figure 5:**
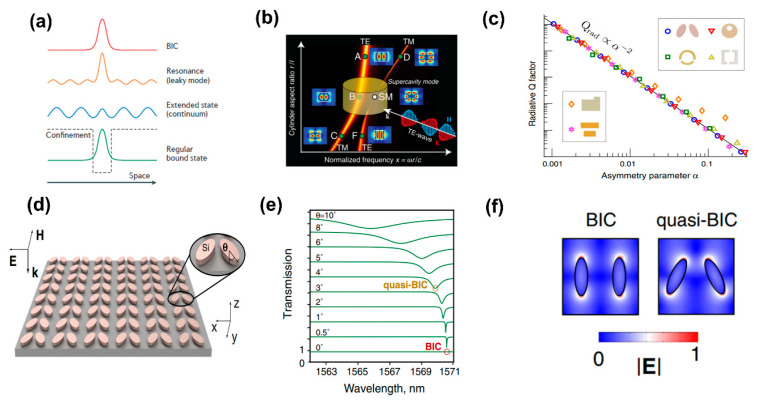
Principle of BIC metasurface. (**a**) General mode profile of BIC. Reproduced with permission from [80]. Copyright © 2016, Springer Nature. (**b**) Example of a high-index dielectric resonator supporting a bound state and strong mode coupling in the continuum. Reproduced with permission from [49]. © 2017 American Physical Society. (**c**) Influence of in-plane asymmetry on the radiative Q factor of quasi-BICs. (**d**) Symmetry breaking in quasi-BIC metasurfaces: the Si asymmetric nanoellipse pairs constituting the metasurface. (**e**) Reliance of the transmission spectra on the angle θ. (**f**) Electric and magnetic field distribution for BICs and quasi-BICs. (**c**–**f**) Reproduced with permission from [79]. © 2018 American Physical Society.

Fundamentally, BIC is a type of mode that exists within the radiation continuum without emitting radiation. The profile of the modes used to depict the BIC state gives a clear indication of the overall picture. Extended states are present throughout a wide range of frequencies (Figure 5a, blue) [80]. Discrete levels of bound states have no emitted flux (Figure 5a, green), and this is the situation with an atom’s bound electrons at negative energies. Because of a confining structure or potential, the bound states are spatially localized (Figure 5a, black dashed line). Resonances that locally mimic a bound state within the continuum can be discovered; however, they really couple to the extended waves and leak out (Figure 5a, orange). BICs are located within the continuum but are fully localized and leak-free (Figure 5a, red).

BICs are characterized as modes with an infinite Q factor and zero line width which do not exhibit coupling to any external radiation channels [80]. The introduction of perturbations can disrupt the existence of pure BICs and lead to the emergence of quasi-BIC modes possessing finite Q factors, which can be detected in the far field. Of the various classifications of BICs, symmetry-protected BICs have received considerable attraction. Plasmonic resonances, by nature, possess relatively low Q factors of approximately 10 [81] as a result of the inherent loss in metals, which has hindered their practical application, as many fields require high levels of spectral selectivity. To enhance the Q factor of a plasmonic metasurface, Aigner et al. [82] used three-dimensional laser nanoprinting technology to fabricate a plasmonic nanofin metasurface that helps multiple symmetry-protected BICs in out-of-plane configurations, reaching unprecedented Q-factors of 84.

Recently, researchers in nanophotonics have explored intense Mie resonances in dielectric nanoparticles exhibiting RIs. The utilization of nanostructured cavities with high refractive indices is motivated by their potential to serve as fundamental components for novel photonic devices with reduced loss and enhanced functionality. Nevertheless, these dielectric resonators exhibit a low quality factor. To compensate for this, a new technique was developed in which high-index dielectric resonators with subwavelengths can sustain supercavity modes (SMs) with high Q factors through tuned parameters that satisfy the BIC condition. A single dielectric cylindrical resonator exhibits substantial interactions between the transverse electric and transverse magnetic modes (Figure 5b) [49]. Due to the near orthogonality of these modes within the resonator, their interference primarily occurs outside of the cavity, thus leading to the manifestation of the BIC and SM [83]. Surprisingly, high Q factors are backed by the mixed polarization SM point. By analyzing the radiative Q factor values of quasi-BICs as a function of the asymmetry parameter for dielectric resonators and PMs with diverse broken-symmetry meta-atoms in their unit cells (Figure 5c). The meta-atoms that make up symmetry-protected BICs hold the key to their resonant behavior, with their in-plane inversion symmetry playing a vital role.

A square array of pairs of Si-nanoellipses was built to acquire insight into BICs in metasurfaces with in-plane asymmetry (Figure 5d) [79]. A phenomenon arises in the spectra as the nanoellipses align parallel to each other, revealing a BIC mode that gradually dissipates as the spectral range is altered (Figure 5e). The in-plane symmetry broken by gradually tilting the ellipses enables a radiation channel and converts the BIC into a quasi-BIC mode with a finite and controllable Q factor. The spectra exhibit a decrease in resonance and a rotation angle-dependent Q factor. The simulated electric field distribution was defined for a duo of nanoellipses, featuring both symmetric and asymmetric configurations (Figure 5f).

The utilization of the BIC concept facilitates the tailoring of the resonant frequency, Q factor, and near-field confinement via the meticulous design of the constituent resonators’ geometry, orientation, and arrangement. This approach provides notable advantages for metasurface biochemical sensing [73,84]. The subsequent sections of this paper elucidate a variety of metaphotonic biosensing applications and explicate their underlying physical mechanisms.

## 3. Applications of Metasurfaces for Biomolecular Detection

The biomolecular metaphotonic sensing using different metasurfaces can be realized by four principal approaches, which include refractometric, surface-enhanced fluorescence, surface-enhanced vibrational spectroscopy, and chiral sensing. The major purpose of this section is to deliver an in-depth understanding of each of these techniques.

### 3.1. Refractometric Sensing

In a refractometric biosensor, analytes bind to resonators generating a localized difference in the RI, which allows for their detection and measurement through the monitoring of the resonant wavelength or the variations in the intensity of the transmission or reflectance [85]. Refractometric sensing does not require chemical adaptations to targets or the ensuing consolidation of additional factors to generate a signal, allowing for label-free and direct detection. Some deciding parameters should reflect concurrently when a metasurface is designed for spectroscopic detection, comprising the resonant wavelength, the resonant bandwidth (or full-width at half-maximum (FWHM)), and the intensity of electric/magnetic near-field enhanced resonances. The comprehensive specifications required for evaluating the sensing efficiency of refractometric biosensors are the sensitivity (S) and figure-of-merit (FOM). Both of them explain the capability of nanostructures to recognize changes in RI in a homogeneous medium. Furthermore, the precision of the quantifiable resonant minimum relates to FOM, which is described as FOM = S/FWHM [86]. The limit of detection (LOD) corresponds to the lowest detectable concentration of an analyte [42,87].

Currently, label-free metaphotonic sensing based on refractometry has emerged as the foremost and most extensively adopted methodology owing to its exceptional performance, ease of use, and economical affordability. In addition, they exhibit the benefits of a compact size and the ability to detect multiple analytical targets simultaneously. Metasurfaces comprising metallic [88] and dielectric [89] nanostructures have the capability to modify light scattering in the sub-wavelength regime, making them highly promising for numerous applications [90].

#### 3.1.1. Refractometric Sensing Based on Plasmonic Metasurfaces

**Figure 6:**
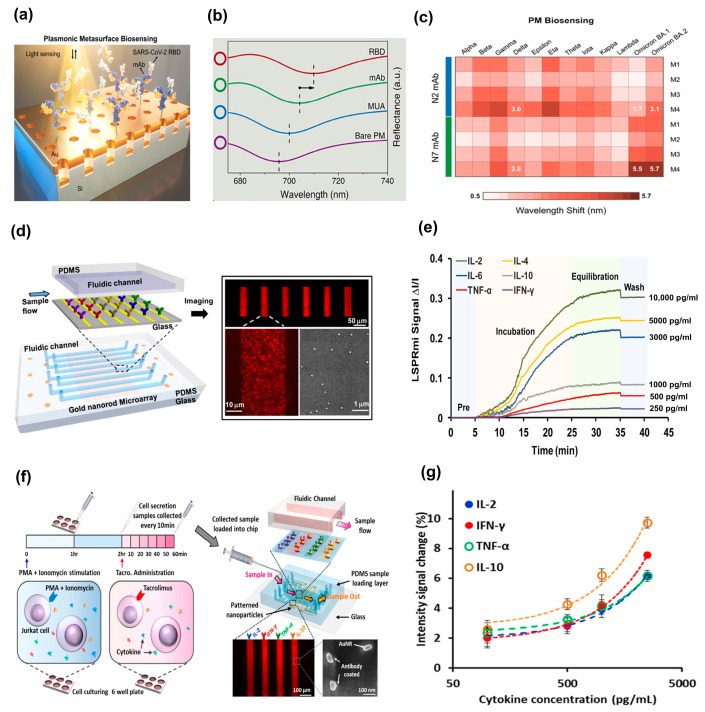
Refractometric sensing based on plasmonic metasurfaces. (**a**) Process of capturing RBD using mAb-functionalized PM and subsequent SPR biosensing. (**b**) Observed spectral shifts owing to the bindings of bare PM, MUA, mAb, and RBD. (**c**) Shifts in resonance wavelength for N2 and N7 mAb. (**a**–**c**) Reproduced with permission from [94]. Copyright © 2023 American Chemical Society. (**d**) Microfluidic-integrated LSPR sensor with multiplexing capability. (**e**) Detecting cytokine biomarkers quantitatively in serum samples. (**d**,**e**) Reproduced with permission from [97]. Copyright © 2015 American Chemical Society. (**f**) T-cell stimulation used in the assay method, dark-field images of four parallel AuNR array patterns, and SEM images of individual AuNR biosensors mounted on glass. (**g**) Standard curves for different cytokines obtained from an LSPR nanoplasmonic biosensor microarray device. (**f**,**g**) Reproduced with permission from [98]. Copyright © 2016 American Chemical Society.

Kretschmann and Reather pioneered the conventional prism-based design in 1968, paving the way for the development of SPR-based sensors [91], whereas Liedberg et al. published the first practical demonstration of utilizing this phenomenon for gas detection and biosensing [92]. The global outbreak of coronavirus (SARS-CoV-2) disease 2019 resulted in the persistent emergence of diverse viral strains. Therefore, the imperative need to discover rapid and reliable detection methods persists to curb the present and prospective episodes of SARS-CoV-2 variants, thereby diminishing the hospitalization and fatality rates among the geriatric population. The biosensors incorporating PMs, fabricated through high-throughput nanofabrication techniques, have garnered immense interest [42,93]. Nonetheless, the previous employment of PM biosensing has been impeded by a tardy affinity detection rate resulting from dynamic sensing and an inadequacy of immunoassay investigations conducted on a wide range of emerging SARS-CoV-2 variants. To surmount these problems, Li Fajun, et al. proposed a high-throughput affinity testing procedure using a label-free PM sensor functionalized with various monoclonal antibodies (mAbs) (Figure 6a) [94]. This platform exhibited their binding attributes to the 12-spike receptor binding domain (RBD) variants of SARS-CoV-2 through prism-based SPR. The spectral shifts of the plasmonic biosensor result from the binding events of 11-mercaptoundecanoic acid (MUA), mAb, and RBD, correspondingly (Figure 6b). Further, the authors assessed the binding abilities of the RBD variant to N2 and N7 (mAb)-functionalized PMs. The optical responses of PMs functionalized with N2 and N7 mAbs are mapped (Figure 6c) against plasmonic modes and different RBD variants at a concentration of 100 ng/mL. The utilization of a high-affinity mAb in conjunction with the substantial surface sensitivity of the plasmonic mode has the potential to significantly enhance the label-free detection of SARS-CoV-2 variants. This research offers a significant breakthrough in the realm of biomolecular interactions and paves the way for the development of cutting-edge POC testing for the detection of emerging SARS-CoV-2 variants in the not-so-distant future.

Recently, the proportion of studies focusing on the clinical applications of plasmonic biosensors has increased [95]. An instance of this trend is demonstrated by Yuan et al. [96], who employed an LSPR biosensor based on silver nanoparticles to detect human epididymis secretory protein 4 obtained from ovarian cancer patients, achieving an LOD of 4 pM. The accurate monitoring of the rapidly changing immunological state during the course of a disease requires the multiplexed analysis of cytokines present in human blood. Chen et al. [97] took a step forward in biosensor technology by developing an integrated platform that utilized LSPR, combining a microarray of gold nanorods with microfluidics (Figure 6d). Their innovative approach enabled the detection of six cytokines in serum with exceptional sensitivity, achieving an LOD < 20 pg/mL (Figure 6e). This biosensor developed by the authors proved to be a reliable and accurate device for cytokine detection, exhibiting a close agreement with commercial enzyme-linked immunosorbent assays.

Moreover, Bo-Ram et al. [98] researched a nanoplasmonic biosensing method for quantitatively assessing the changes caused by an immunosuppressive medication used to treat T-cell-mediated illnesses. A six-well culture plate containing Jurkat cells, a popular human leukemia cell line used to study the signaling pathways of T-cell receptors, was tested (Figure 6f, left). A bottom glass layer and a top polydimethylsiloxane (PDMS) layer make up the LSPR biosensor chip that was employed (Figure 6f, right). On a single chip, this gadget design produced a total of 120 sensing dots. Each of the four gold nanorod strips (AuNRs) was effectively functional with an anticytokine antibody targeted against interleukin-2 (IL-2), interferon-gamma (IFN-γ), tumor-necrosis factor alpha (TNF-α), and interleukin-10 (IL-10). To confirm that unique antibody-conjugated AuNRs were evenly dispersed over the glass substrate with an interparticle spacing > 100 nm, scanning electron microscopy (SEM) was performed. It was essential that the interparticle spacing was sufficiently large to prevent plasmonic coupling between neighboring particles, which could reduce the sensitivity of analyte detection [97]. Additionally, prior to $\left(I_{0}\right)$ and following ($I_{0}+∆I)$ sample incubation, the intensity values of the LSPR sensing sites were measured. Typical curves showing the fractional intensity shift $\frac{∆I}{I_{0}}$ variation with cytokine concentrations were plotted (Figure 6g).

#### 3.1.2. Refractometric Sensing Based on Dielectric Metasurfaces

**Figure 7:**
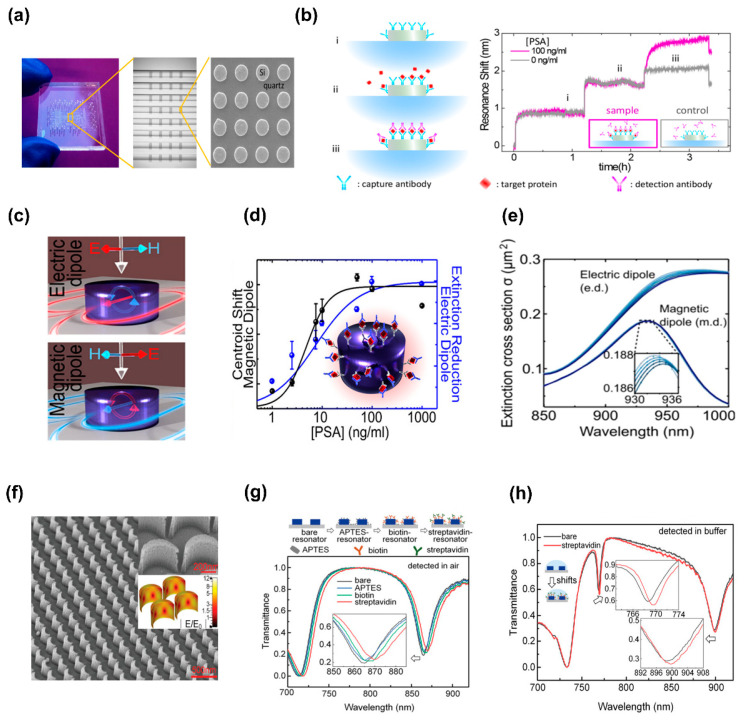
Refractometric sensing based on dielectric metasurfaces. (**a**) Silicon-based on-a-chip biosensing using a quartz substrate. (**b**) Diagram of the sensing protocol (left) and the change in the nanodisk resonance as the sandwich experiment progressed (right). (**a**,**b**) Reproduced with permission from [53]. Copyright © 2017 American Chemical Society. (**c**) Si-NCs metasurface assists electrical and magnetic dipolar resonances. (**d**) Illustration of the processes in the sandwich assay for the detection of antigens. (**e**) Si-NC array’s extinction cross-section: electric and magnetic dipolar elements. (**c**–**e**) Reproduced with permission from [100]. Copyright © 2019 American Chemical Society. (**f**) SEM micrograph of the manufactured Si crescent metasurface and a simulated near-field distribution of the resonator (inset). (**g**) Sensor transmittance spectra as a function of the molecule immobilization stages. (**h**) Transmittance spectra of the immobilized biotin-streptavidin sensor and the bare sensor in the buffer. (**f**–**h**) Reproduced with permission from [102]. © 2021 The Authors.

The robust Mie-type resonances in dielectric metasurfaces offer near-field enhancement that induces obvious light–matter interactions with analytes around the resonator areas. Depending on the metasurface design tunability, the resonant behavior might be sensitive or responsive to the optical characterization of the surrounding medium. Because of low losses of intrinsic optical absorption inside the dielectric nanoresonators, a high Q factor and a considerable enhancement of electric/magnetic near-fields can be realized [36]. The reduction in localized heat on illumination at their resonance wavelengths was also observed [76]. Refractometric label-free sensing originated from a low-loss dielectric microstructure, and the higher-level Mie resonances called whispering-gallery modes show Q factors with values of over 106 for simple configurations, such as Si microspheres, disks, and ring waveguides [99]. Moving from the microscale to the nanoscale, dielectric nanostructures exhibit more humble Q values, enabling more broadband activity in certain low-loss circumstances. Regarding simple dielectric resonant architecture, the wavelength shifts related to analyte binding are commonly smaller than those of plasmonic nanostructures. However, recently, researchers have utilized biotin-functionalized Si disks to detect the low concentration of streptavidin in buffers down to 5 ng mL^−1^ in the near-infrared range [52]. The biosensing reveals a resonance at approximately 1488 nm, with a Q-factor of 25, which allows for the detection of low concentrations of streptavidin to sub ng mL^−1^ due to robust, optically induced magnetic resonances from Si nanodisks. In addition, Yavas et al. optimized the fabricated Si nanoresonators with near-IR wavelengths [53] integrated with state-of-the-art microfluidic devices to demonstrate bulk RI biosensing along with prostate-specific antigen (PSA) detection for a protein cancer marker in human serum. The microfluidic configuration including micromechanical valves, near-image independent sensing channels, and an SEM figure of the Si nanodisk on a quartz substrate is shown in Figure 7a. The platform was equipped with a stage scanning scheme for the sequential acquisition of data from multiple spatial points in the sensing chip. The enhanced efficient RI in the sensor proximity resulting from the binding steps generates red-shifts in the resonances of the nanodisk arrays, permitting the precise monitoring of molecular adsorption and binding kinetics. The biomolecule detection process operates on a conventional sandwich assay methodology, where the target protein is entrapped between two distinctive antibodies. One of the antibodies, immobilized on the Si surface, functions as a capture antibody, whereas the other antibody is introduced in the solution and acts as a detection antibody (Figure 7b, left). Moreover, the authors demonstrated sensing of the PSA cancer biomarker in a buffer with an LOD down to 1.6 ng mL^−1^ (Figure 7b, right).

Utilizing approaches to suppressing radiative coupling into the continuum can increase the RI sensitivity of dielectric nanoresonators, thus narrowing the resonance linewidth; however, these benefits come with the damage to the operating bandwidth reduction. Distinct contributions of the lowest-level ED and MD resonances in Si nanocylinder arrays correlate with both extinction decline and a resonant red shift (Figure 7c) [100]. The LOD for the PSA protein cancer biomarker assessed by the extinction decrease was recorded to be 0.83 ng mL^−1^, superior to that of resonance shift-based analysis (1.55 ng mL^−1^) (Figure 7d). Such difference in detection can be attributed to the high interaction between the electric field and the surrounding environment, whereas the MD modes are highly restricted in the resonator, resulting in less sensitivity to surface modifications. The interesting thing is that, via dividing the extinction cross-section into ED and MD modes (Figure 7e), the underlying principles behind the resonance shift and the decrease in the extinction can be revealed. This indicates that supplementary scrutiny of intensity interrogation approaches is indispensable in realizing high-performance spectrometer-less biosensor platforms for POC devices.

Numerous biological and medical bioassays in real time are performed in a fluidic buffer media. This thing reduces the RI discrepancy between surface-based bonded/adsorbed molecules and the dielectric surroundings compared to air measurements, decreasing the obtainable sensitivity [101]. To address this concern, a high-Q higher-order resonance in a BIC-inspired crescent metasurface has been investigated to achieve a higher spectral resolution and enable the sensing of ultrathin layers of biomolecules in solutions [102]. A high-resolution scanning electronic microscopy side and magnified view of the dielectric crescent metasurface with a highly homogeneous structure are shown in Figure 7f and the inset (top right), respectively. The numerical simulation of quasi-BIC resonance in the metasurface performed using the commercial electromagnetic solver CST Microwave Studio Suite indicates that the model of the crescent shows localized near-field enhancement around the surface (Figure 7f, inset below the right). For the detection of a biomolecular layer, a high-affinity biotin-streptavidin biological test was selected. There is a clear red-shift at each step of surface modification, as the detection was performed in air (Figure 7g). To evaluate the potentiality of higher-order resonances in streptavidin protein binding under the buffer media (n~1.333), a total resonant shift in the buffer was tracked (Figure 7h). The change in the resonance dip is more apparent from the higher order than in the fundamental one because of the narrower FWHM. The resonance shifts monitored in the buffer are smaller than those in air; this matter can be an encouragement for carrying out a sandwich bioassay [56].

### 3.2. Surface-Enhanced Fluorescence Sensing

Surface-enhanced fluorescence (SEF) is promptly becoming one of the main spectroscopic techniques for detecting a bunch of biomolecules and biomarkers. Recently, metallic structures have been extensively studied for fluorescence-based sensing with the aim of attaining increased sensitivity and lower detection limits. The resonance frequency of resonators can be tuned in numerous ways [103] to permit the simultaneous augmentation of fluorophore excitation and emission while preserving robust near-field enhancements [104,105]. An effective method of circumventing the diffraction limit and localized electromagnetic energy into nanoscale spatial dimensions is to use plasmonic metal nanostructures serving as optical antennas [106,107]. The interactions between a single quantum emitter and its surrounding photonic environment are also significantly improved by these optical antennas [108,109,110], resulting in a colossal luminescence enhancement [111,112] and directional emission control [113,114]. Regarding the high micromolar concentrations necessary to achieve the physiological parameters that are biologically relevant, all these characteristics make optical antennas the perfect choice for the ultrasensitive biodetection of individual molecules.

**Figure 8:**
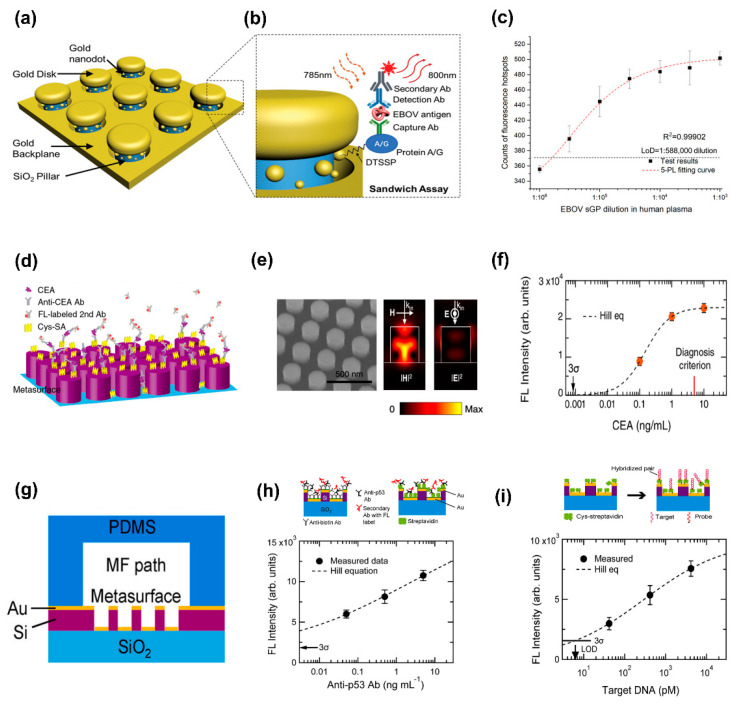
Surface-enhanced fluorescence sensing. (**a**) 3D plasmonic nanoantennas for sensing Ebola virus antigens. (**b**) Magnified view of a single nanopillar structure and EBOV sandwich assay on a chip. (**c**) Calibration curve for low concentrations of the soluble glycoprotein antigen on nanoantenna arrays. (**a**–**c**) Reproduced with permission from [56]. © 2019 WILEY-VCH. (**d**) Detection test of CEA on the all-dielectric metasurface. (**e**) SEM micrograph acquired at a 30° oblique angle and EM-field intensities of |H|^2^ and |E|^2^ at 559.3 nm (numerically estimated). (**f**) Fluorescence-intensity scheme for the CEA concentration. (**d**–**f**) Reproduced with permission from [118]. Copyright © 2020 American Chemical Society. (**g**) Illustration of a metasurface sensor chip including hybrid architectures comprising Si waveguides and gold nanostructures (stacked complementary). (**h**) Indirect detection of anti-p53 Abs without an FL label. (**i**) Sensing of oligos synthesized complementary DNA to RNA of SARS-CoV-2. (**g**–**i**) Reproduced with permission from [119]. © 2021 Elsevier.

A plasmonic sensor, comprising a silicon oxide (SiO_2_) nanopillar array with gold nanodisks positioned atop each pillar and gold nanodots on the walls of the pillars, fabricated on a gold surface (Figure 8a) [56], has exhibited the ability to detect pathogens with exceptional sensitivity. The early diagnosis and quarantine of diseases are crucial for the effective management of severe and potentially lethal outbreaks. Consequently, the sensitive detection of pathogens is of utmost importance in this regard. Utilizing a sandwich assay protocol (Figure 8b), this 3D nanoantenna sensor successfully quantified the soluble glycoprotein (s-GP) of the Ebola virus (EBOV) in human plasma, demonstrating an impressive sensitivity level in detecting s-GP at a concentration as low as 220 fg mL^−1^ (Figure 8c). The researchers conducted additional tests and confirmed that the sensor was capable of detecting sGP in human plasma samples that were spiked with sGP at a concentration twice that of the detection limit, without compromising the sensitivity level achieved in the original test.

All-dielectric metasurfaces with low-loss high-RI materials eliminate detrimental ohmic losses and quenching effects from metallic structures when stimulated at wavelengths above their bandgap [115]. All-dielectric metasurfaces without metal deposition have an advantage over plasmonic counterparts [116,117] because of the comparably easier manufacturing. As a result, low-cost devices are anticipated in real-world applications. Reusability by washing the metasurface substrates is another benefit of all-dielectric metasurfaces; this asset will help to reduce operating costs significantly. To leverage these benefits, all-dielectric metasurfaces have been extensively used for fluorescence-based biomolecular detection. Iwanaga et al., for instance, developed the sandwich assay methodology using an all-dielectric Si metasurface to detect antibodies and antigens with a high sensitivity and enhanced detection limit [118]. A sandwich-assay design was used for carcinoembryonic antigen (CEA) detection (Figure 8d). First, the silicon-on-insulator (SOI)-nanorod metasurface was fabricated on an SOI substrate with a middle buried oxide (BOX) layer of 375 nm and a top SOI layer of 200 nm. Then, cys-streptavidin was immobilized on the SOI-nanorod metasurface before serving as the binding sites. The reaction mixture contained CEA and anti-CEA Ab without a biotin tag. Subsequently, the secondary fluorescence (FL)-labeled antibodies (Abs) were flown through at a concentration of 100 ng/mL to partially bind with the anti-CEA Ab. Because of the polyclonality of the secondary Abs, multiple binding between the secondary Abs is possible (Figure 8d), and this results in FL amplification. They discovered that the electric field intensity is maximal at the resonators’ outermost surface (Figure 8e), which causes a significant increase in fluorescence. The SEM image captured at 30° is shown in Figure 8e (left side); the scale bar is 500 nm. Resonant electromagnetic field distributions: the magnetic- and electric-field intensities, |H|^2^ and |E|^2^, respectively, on the linear scale, are illustrated in Figure 8e (middle and right), which exhibit the resonance linked to the FL-intensity enhancement in a wavelength range of 560–600 nm. Furthermore, CEA detection was effectively carried out much below the clinical diagnostic level of 5 ng mL^−1^ and with an LOD of 0.85 pg mL^−1^ (Figure 8f). With detection periods of less than 90 min, this sensing technology provides superior sensitivity and throughput compared to conventional ELISA.

Various investigations into FL-enhancing effects in plasmonic platforms have been conducted to date; however, the applicability of FL-enhanced plasmonic biosensors in identifying real-world illness indicators is rarely demonstrated. Masanobu Iwanaga [119] showed that plasmon-photon-hybrid metasurfaces can be used as effective biosensors for a variety of targets, including DNA and Abs. The structure consisted of stacked complementary (SC) gold nanostructures and a Si slab waveguide with a nanohole array. A segment parallel to the microfluidic (MF) path is depicted in Figure 8g. Experimental evidence proves significant FL-intensity improvement (over 2000 folds) for flat Si wafers [117]. This noticeable improvement is possible by complete light absorption control, the prevention of FL-quenching energy transfer, and high emittance at FL wavelengths [116]. Therefore, the FL-enhancing effects are highly consistent and repeatable, and the SC metasurfaces do not solely rely on local electric-field augmentation. Even though it is generally accepted that strong local electric fields (hot spots) are vital in obtaining noticeable FL enhancement, hot spots invariably lead to irregular responses and negatively impact the repeatability of the enhanced signals. Target anti-p53 Ab (cancer indicators for colorectal cancer in the early stage) immobilization configurations is illustrated in Figure 8h (top, right, and left). The binding molecules used in the two configurations, rabbit-polyclonal anti-biotin Ab (Figure 8h, top right) and streptavidin (Figure 8h, top left), are distinct from one another. The measured FL intensity is displayed with closed circles and error bars, and the Hill-equation fitted curve is shown as a dashed line in Figure 8h (bottom). The data are almost scaled by a power of 0.224 in the observed range, indicating that quantitative analysis is possible with good sensitivity, even at a low target concentration (50 pg mL^−1^). They examined the ability of the metasurface biosensors to detect molecules of nucleic acids, including the discovery of oligonucleotide cDNAs and the RNA of SARS-CoV-2. The immobilization structure is shown in Figure 8i (top). A horizontal bar represents the 3σ (σ: standard deviation) level (Figure 8i, bottom), which was calculated using no target data. The determined LOD for the cDNA target was 6.3 pM. The LOD in direct detection methods is minimal; however, it is larger than that obtained from amplification-based methods such as RT-PCR. Given that the DNAs are short, it is possible that SC metasurfaces could serve as a platform for microRNA (miRNA) detection. After comparing the LOD from direct sensing techniques in terms of the dynamic ranges [120,121,122], it can be stated that the scaled ranges identified the target concentrations at 100 pM; however, no obvious report has presented a calibrated detection curve below 100 pM. The series of scaled data is shown in Figure 8i (bottom); the assessed LOD is assessed to be 6.3 pM. The SC metasurface is thus one of the most effective plasmonic platforms for directly detecting nucleic acids and may provide quantitative detection for targets at low concentrations (100 pM). Compared to the SC metasurfaces’ hole-array structure, the protuberant structure probably contributes to the improved performance. Furthermore, Romano et al. showed that both SEF and refractometric sensing based on the underlying photonic quasi-BICs can monitor the spatially variable surface RI of living cells cultivated on an all-dielectric photonic crystal slab (PhCS) [73]. It was demonstrated that the emission of the emitter was amplified by two orders of magnitude by the first intense resonance near-fields. The specimen’s spatially variable RI was mapped simultaneously on the PhCS via hyperspectral refractometric sensing, which made use of the Fano interference between the second mode and the fluorescence emission. A correlative imaging platform realized by this dual sensing technique can be used in a variety of domains, including surface cell research, molecular interactions, and chemical processes.

Although multiplexed tests with a high throughput can be performed using refractometry and SEF-based sensing, they are still unable to extract the entire chemical-specific information related to biomolecular surface binding in terms of both time and space. This gap can be filled by surface-enhanced vibrational spectroscopy.

### 3.3. Surface-Enhanced Vibrational Spectroscopy

Vibrational spectroscopy is a different approach to refractometric and surface-enhanced fluorescence for detecting biomolecules, which not only enables the identification of surface-bound molecules but also offers chemically specific details about each analyte. Raman and Infrared (IR) absorption spectroscopies, for example, provide label-free sensing and monitoring of chemical reactions and the kinetics of interactions between different analytes. Raman spectroscopy uses the inelastic scattering of photons to examine molecular vibrations, whereas IR spectroscopy employs measurements of light absorption to obtain chemical information. However, because the target molecules have extremely low absorption cross-sections, it is necessary to use a copious number of analytes or powerful laser sources, which increases complicated technologies, can cause molecule degradation, and restricts the detection of the number of analytes. Surface-enhanced vibrational spectroscopy methods, especially surface-enhanced Raman spectroscopy (SERS) and surface-enhanced IR (SEIRA) spectroscopy, which rely on amplifying molecular vibrations through the interaction with nanostructured surfaces, have been developed to overcome these limitations.

#### 3.3.1. Surface-Enhanced Raman Spectroscopy

When molecules are adsorbed onto corrugated metal surfaces, such as silver or gold nanoparticles, they dramatically increase the amount of inelastic light scattering. This process is referred to as SERS [123]. SERS is considered a powerful tool in material and life sciences, owing to its intrinsic features (i.e., fingerprint recognition capabilities and high sensitivity), cost-effectiveness, and user-friendly operation. Raman spectroscopy for biosensing has several benefits, including the elimination of an undesired background in aqueous samples. Most SERS substrates have a random spatial distribution of intense “hot spots”, making it difficult to forecast where these spots will be for continuous activation. Importantly, the SERS EF rapidly decreases over a few nanometers from the metal surface, which reduces the applicability of SERS for probing massive biomacromolecules or complexes. Large proteins also make the intrinsic SERS detection of protein molecules or variations in their orientation or shape challenging. Weak Raman signals can also be easily concealed by autofluorescence. The intensities of the enhanced surface Raman scattering are estimated as [124]:

(3) $$I_{SERS}=I_{0}{\left|\frac{E(\omega_{ext})E(\omega_{det})}{E_{0}(\omega_{ext})E_{0}(\omega_{det})}\right|}^{2},$$

where E and E_0_ are the electric field strengths before and after the amplification of the plasmonic structure, respectively. $\omega_{ext}$ and $\omega_{det}$ are the excitation and detection frequencies, respectively. The EF is denoted by the formula ${EF}_{\omega }={\left|\frac{E(\omega )}{E_{0}(\omega )}\right|}^{2}$. The Raman EF can be written as ${EF}_{SERS}=EF\left(\omega_{ext}\right)EF{(\omega }_{det})$. The resonance of the maximally amplified SERS signal happens between the wavelength of excitation and Raman scattering [125]. The electric field has a direct impact on the spatial homogeneity of ${EF}_{SERS}$. High spatially and uniformly distributed field enhancement is required for accurate results.

**Figure 9:**
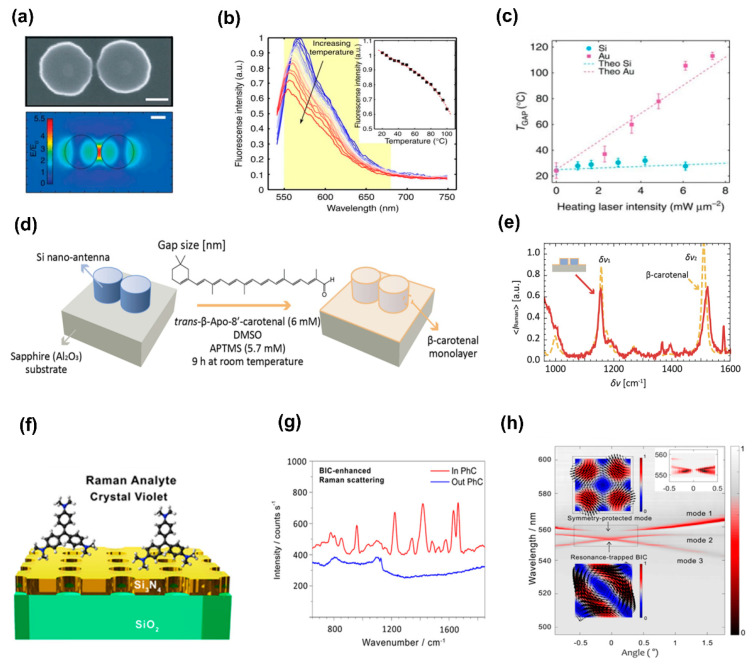
Sensing based on surface-enhanced Raman spectroscopy. (**a**) Dielectric metasurface using Si−dimer nanoantennas employed to improve the Raman scattering signal of polymer films. (**b**) Nile Red emission spectra at varying temperatures with the detection range marked (550–680 nm). (**c**) Temperature measurement in the gap for gold (magenta) and Si (cyan) nanoantennas as a function of the heating laser’s intensity at 860 nm. (**a**–**c**) Reproduced with permission from [76]. Copyright © 2015, The Author(s). (**d**) Si-dimer nanoantenna-based all-dielectric metasurface functionalized β-carotenal on an alumina substrate. (**e**) Enhancement of the SERS signal of the β-carotenal monolayer. (**d**,**e**) Reproduced with permission from [54]. Copyright © 2018 American Chemical Society. (**f**) All-dielectric metasurface based on silicon nitride nanopores provide for the quasi-BIC mode. (**g**) Raman scattering spectra of the internal and external crystal violet molecule of the photonic crystal metasurface. (**h**) Drawing of the dispersion bands derived from TE transmission spectra. (**f**–**h**) Reproduced with permission from [138]. Copyright © 2018 American Chemical Society.

The indirect label [126] and the direct label-free approach [127] are two of the most often used SERS strategies. In label-mediated SERS, molecules of interest are detected indirectly using pre-functionalized SERS tags that are created with the intention of following the target analyte molecules for quantification and distinction. Conversely, label-free SERS techniques directly measure the Raman signals of the target molecules, enabling in-the-moment monitoring that is contactless, non-invasive, and non-destructive, particularly for biological systems [128,129]. SERS has traditionally been implemented using metallic nanostructures [130,131] and metasurfaces [132,133], which offer potent near-field enhancement. Even if the signals are enhanced significantly by their surface improvement, the metasurface still requires a high laser intensity and tight light focusing. This results in significant heating in metal-based metasurfaces, which can damage the biomolecules [134], modify the local RI [135], or even deform the metasurface itself [136]. SERS measurements using metal-based metasurfaces are therefore subject to reproducibility problems [128]. For Raman spectroscopy, dielectric metasurfaces have recently become an alternative to plasmonic counterparts. Reduction in heat generation is possible because of the nearly zero absorption losses of the materials constituting dielectric metasurfaces, and significant light confinement is produced by the high RI. Effective nanoantennas with low light-to-heat conversion rates fabricated on a unique nanophotonic platform based on dielectric nanostructures, according to research by Caldarola et al. [76] (e.g., a metasurface made of a Si dimer array on an SOI substrate), generated substantial levels of surface-enhanced fluorescence and surface-enhanced Raman scattering with the slight temperature rise of the hot spots and surrounding areas. The diameter, height, and form of a single Si-dimer nanoantenna with a 20 nm gap between the two disks are shown in Figure 9a (top). The calculated near-field distribution is depicted in Figure 9a (bottom), which demonstrates the optical antenna performance around a Si dimer stimulated at the resonance (*λ* = 860 nm). The dipolar resonance of the structure causes the strongest field enhancement in the gap, achieving values close to 5.5 for E/E_0_. Single-molecule SERS detection should be possible [137] given that theoretical models predict (E/E_0_)^4^ rises of up to 10^6^ (E/E_0_ = 32) for Si dimers with gaps as small as 4 nm [75]. Caldarola et al. employed a thermal imaging technique that integrates molecular thermometry with a diffraction-limited spatial resolution, achieving a resolution of approximately 370 nm, to study the temperature characteristics of nanoantennas. The Nile Red fluorescence profile is acutely sensitive to changes in temperature, allowing it to serve as a probe for detecting local temperature fluctuations (Figure 9b). The relationship between the gap temperature and heating laser power is shown in Figure 8c. The temperature rise in the gap for the gold nanoantennas surpasses 80 °C, contrary to expectations, and is extremely modest for the Si nanoantennas. This finding is consistent with theoretical hypotheses, and it was shown that there is good agreement between these obtained gap temperatures and those retrieved from numerical computations (dashed lines). The authors also determined a heating slope ratio (Au/Si) of 17.6 (Figure 9c). Further, numerical studies of the temperature rise for heating laser intensities up to 120 mW/m^2^ revealed that the gold nanoantennas would increase the gap temperature above 1200 °C, whilst the Si nanoantennas would only see a temperature increase not more than 100 °C.

Javier Cambiasso et al. fabricated a Si dimer metasurface on a sapphire substrate to identify a self-assembled monolayer of β-carotenal molecules [54]. As illustrated in the scheme (Figure 9d), they created a Schiff base of -carotenal and (3-aminopropyl) trimethoxysilane (APTMS) to covalently conjugate the β-carotenal. For a homogeneously covered sample, bare Si dimers on sapphire were incubated in a solution of β-carotenal and APTMS for nine hours. Here, a diagram of the measured enhancement factors and the estimated values derived from simulations are shown in Figure 8e. For the peak at δv1 = 1154 cm^−1^, the enhancement factor is 1720 ± 300. The enhancement factor for δv2 = 1522 cm^−1^ is 1380 ± 200, and it is also demonstrated that this value coincides with the simulated value of 1420.

A BIC-type all-dielectric metasurface with silicon nitride (Si_3_N_4_) nanopores was investigated to improve the capacity of nanophotonics in enhancing the SERS signal [138]; a 103-fold increase in the Raman signal of molecules of crystal violet (CV) dye (Figure 9f) was detected with this metasurface. This CV dye was selected because its fluorescence emission spectrum and Raman fingerprint are different. Therefore, unlike the Raman spectrum of R6G, the Raman scattering signal of CV does not entirely embed in a high-fluorescence background. The researchers inclined the sample angle by approximately one degree for resonance matching to increase the signal amplification. The BIC-enhanced Raman spectrum excited on the photonic crystal metasurface (PhCM) is illustrated in Figure 9g. The unpatterned Si_3_N_4_ showed no discernible Raman scattering. Moreover, the transmission TE band of a 54 nm thick PhCM is displayed in Figure 9h. A very small dip in the transmission occurs when a mode develops at a particular angle; this dip gradually disappears as it gets closer to the diverging Q-factor BIC mode [139,140]. Three dispersion bands are experimentally seen in their PhC metasurface (modes 1–3) (Figure 9h). A single degenerate mode is represented by the first one (symmetry-protected mode). The mode should have an arbitrarily large Q-factor at 0°. Away from the typical incidence, its Q-factor substantially diminishes (Γ point = 0°). The top inset (Figure 9h) displays the anticipated symmetry-protected BIC.

Metal-based antennas continue to offer higher field improvements and better SERS signals, despite early efforts toward dielectric metasurfaces for SERS. However, it is possible to further improve dielectric metasurfaces by refining the designs of the meta-atoms that allow for high-Q resonances and the greatest near-field enhancements beyond the resonator volume. This helps biomolecules access the near-fields, resulting in a stronger SERS signal. Additionally, a variety of surface functionalization techniques for analyte bonding [141] and chemical enhancement [142] are possible because of the vast range of readily available dielectric materials.

#### 3.3.2. Surface-Enhanced Infrared Absorption Spectroscopy

Because all of the fundamental molecules that make up life carry a wealth of vibrational information, SEIRA is especially well suited for researching biological systems [143]. It is possible to estimate the strength of the optical absorption signature obtained from an analyte using Beer’s law, which requires an exponential decline in the vibrational signal with analyte thickness [144]. Therefore, when examining analytes placed on a substrate at few-molecule levels, bulk absorption spectroscopy encounters considerable sensitivity constraints. The use of resonant nanoantennas and specifically nanophotonics can overcome this limitation by bridging the length scale difference between IR wavelengths (order of microns) and molecular analyte dimensions (the order of nanometers) [145,146]. These antenna configurations concentrate the incident light into electromagnetic field hotspots, resulting in strong light–matter interaction and allowing for the identification of otherwise weak absorption fingerprints down to protein monolayers [145,147]. Numerous sensing applications in a variety of disciplines, including pharmaceutical, environmental monitoring, and human health, have been made possible by SEIRA [148]. Furthermore, infrared spectroscopy’s non-invasive and label-free properties enable researchers to monitor in situ molecular interaction kinetics in situ in complex bioassays or processes involving biological membranes in real time. The geometries, material selection, and spatial arrangement of the nanostructured antenna elements play a key role in determining the sensitivity and, thus, the LOD of SEIRA.

**Figure 10:**
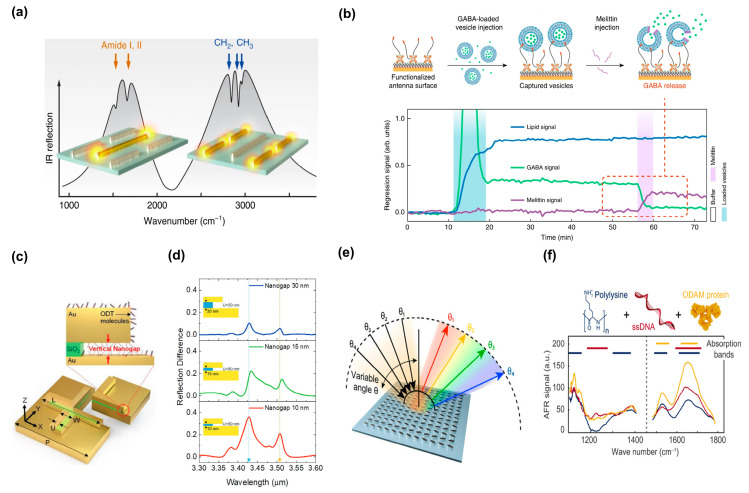
Sensing based on surface-enhanced infrared absorption. (**a**) Resonance positions of the antenna designed to overlap with amide I, II, CH2, and CH3 absorption bands, enabling the detection and enhancement of protein- and lipid-induced absorption changes concurrently. (**b**) Identification of melittin-induced membrane rupturing and release of the neurotransmitter gamma-Aminobutyric acid (GABA) from loaded vesicles. (**a**,**b**) Reproduced with permission from [149]. Copyright © 2018, The Author(s). (**c**) Schematic of the ODT monolayer-coated metamaterial absorber (MA) with a vertical nanogap edge section. (**d**) Top, middle, and bottom panels with vertical nanogap structures at 30 nm, 15 nm, and 10 nm, respectively, display the determined SEIRA reflection difference signal for the MA. (**c**,**d**) Reproduced with permission from [152]. © 2021 The Authors. (**e**) Ge resonators arranged in zigzag arrays on a CaF_2_ substrate to form a high-Q-factor dielectric metasurface. (**f**) Extensive spectrum coverage of the angle-multiplexed approach permits the chemically precise fingerprint identification of a variety of analytes in a bioassay comprising interactions of polylysine, DNA, and ODAM protein molecules. (**e**,**f**) Reproduced with permission from [154]. Copyright © 2019 The Authors.

Complex interactions between lipid membranes and proteins are vital for the operation of a wide range of biological activities. It is necessary to distinguish the constituent biomolecular species and follow each one’s individual time progression without using labels to comprehend such dynamic processes. By utilizing a multi-resonant mid-IR metasurface, Rodrigo et al. investigated the real-time monitoring of lipid–protein systems in an aqueous environment [149]. Their study proposed a metasurface featuring two sets of gold nanodipoles, each independently modulating the electromagnetic response to enable the simultaneous enhancement and detection of absorption changes associated with lipids and proteins (Figure 10a). To capture intact cargo-filled lipid vesicles with a diameter of 50–70 nm on the metasurface, they first designed a surface functionalization approach. This allowed for the surveillance of a biological process (Figure 10b, top). This was accomplished by employing biotinylated thiols to functionalize the gold antenna surface. Next, streptavidin was attached and used to bind the biotin-PEG-cholesterol vesicle tethers. The release of the cargo was initiated by the melittin-induced perforation of the vesicle membrane in the second phase, which involved capturing Gamma-aminobutyric acid (GABA)-filled vesicles on the functionalized metasurface. Moreover, time-resolved linear regression signals were used to efficiently differentiate the three biomolecular components of the experiment: lipid, melittin, and GABA (Figure 10b, bottom). This result illustrates the usefulness of this method in biological systems with more than two analytes, if their IR spectra are adequately distinct and linearly independent over the detection bands of the metasurface. This work demonstrate that SEIRA-based biosensors can aid in the study of significant classes of lipid vesicles, such as synaptic vesicles in neurological illnesses, exosomes in cancer, and drug release processes from liposomes.

Large-area patterning is difficult and expensive. Most resonant SEIRA devices utilize near-field enhancement caused in the in-plane nanometer-sized gap, which necessitates high-precision manufacturing tools, including e-beam lithography. The creation and analysis of many pattern arrays require a pricey high-precision fabrication technique, which limits the use of this technology. In addition to the other suggested structural platforms, metamaterial absorbers (MAs) have also shown impressive SEIRA signal augmentation. MAs with a metal–insulator–metal configuration are commonly built with a dielectric spacer layer placed between an optically thick metallic back plane and a structured metallic top nanoantenna, which enables perfect absorption at a certain wavelength by entirely suppressing light transmission and reflection [150,151]. Further improving SEIRA signals requires increasing near-field coupling with the sample molecules’ IR absorption mode. To mitigate this issue, Hwang et al. created a vertical nanogap by isotropic dry etching an ultrathin dielectric spacer placed between the top nanoantenna fabricated by nanoimprint lithography (NIL) and the bottom gold plane (Figure 10c) [152]. This increased the effective interaction area of the light wave and molecules, creating an extremely strong electromagnetic field between the top and bottom metal layers. The low-cost production method suggested in this study can create a vertical nanogap of 10 nm over a sizable region. To assess and validate the octadecanethiol (ODT) monolayer’s sensing performance, theoretical and experimental analyses of MAs with three different vertical nanogap thicknesses were conducted. A strong reflection difference SEIRA signal of 36% was produced in the smallest 10 nm thick vertical nanogap at a wavelength of 3.5 µm (Figure 10d).

Analogous to SERS, metal-based metasurfaces have mostly been used in SEIRA measurements. Even in the IR spectral region, the performance of plasmonic antennas, which are excellent for signal augmentation, is fundamentally constrained by the Ohmic losses. This greatly widens their resonances and FWHM compared to the molecular absorption bands. Thus, a large spectroscopic apparatus is typically needed to detect IR absorption bands and extract chemical information [145]. Dielectric materials enable the attainment of higher Q-factor resonances in the mid-IR, where the resonance FWHM is narrower than the molecular absorption bands of bioanalytes. This characteristic makes the molecule absorption bands targeted with improved spectral selectivity while preserving high sensitivity [153]. A pixelated all-dielectric metasurface with ultrasharp resonances has been proposed by Tittl et al. for imaging-based molecular identification [55]. The anisotropic hydrogenated amorphous silicon (a-Si:H) resonators constituting the metasurface are arranged in zigzag arrays on the magnesium fluoride (MgF_2_) substrate, and the resonant frequency is tuned to cell size. This method’s simple resonance tuning over a wide spectral range via geometrical scaling, which targets the mid-IR fingerprint range of 800 to 4000 cm^−1^ while keeping Q factors above 100, is a significant benefit. It is fascinating to note that the absorbance of the surface-bound analytes can be easily retrieved from the total reflectance data, without the need for adjustable light sources or expensive spectroscopy equipment, using this 2D-pixelated metasurface.

Moreover, Leitis et al. have also tuned highly surface-sensitive and spectrally sharp resonances using a single BIC-based metasurface and incidence angle of mid-IR light for protein detection over a broad spectrum spanning from 1100 to 1800 cm^−1^ (Figure 10e) [154]. The most near-field improvement is realized at the tips of the ellipse-shaped meta-atoms used in both works, where the nearby bioanalytes can interact with the light. This approach only requires a broadband light source and detector to achieve high sensitivity chemical fingerprint detection. This technique combines the chemical specificity of IR spectroscopy with the device simplicity of angle-scanning refractometric sensing. The illustration of this metasurface-equipped system for detecting polylysine, single-stranded DNA, and human odontogenic ameloblast-associated proteins (ODAM) is shown in Figure 10f. Single-stranded DNA molecules were incubated on the metasurface following polylysine deposition and a deionized water rinse. Electrostatic interaction causes the negatively charged DNA aptamers to bind to the positively charged polylysine. The increases in the specific absorption signal of the bound aptamer molecules at 1235 and 1650 cm^−1^ are in good agreement with the single-stranded DNA absorbance data from the literature [155]. Strong signals appearing at the unique amide I and amide II absorption bands at 1540 and 1660 cm^−1^ were used to detect the binding of the ODAM protein to the single-stranded DNA aptamers, which was the last phase of the bioassay (Figure 10f). The angle-multiplexed detection method demonstrates a strong signal-to-noise ratio, enabling the detection of submonolayer analyte molecule quantities with a limit of 3000 molecules/m^2^, which is equivalent to a surface mass sensitivity of 0.27 pg/mm^2^. Because molecular fingerprint data can be obtained using broadband light sources and detectors, such high-Q metaphotonic platforms show significant promise for the development of spectrometer-free POC sensing devices.

### 3.4. Chiral Sensing

Chirality is the property of objects that prevents them from being superimposed by their mirror image after a single rotation or translation. A chiral object and its mirror counterpart, also known as an enantiomer, have almost identical physical qualities; yet, when exposed to circularly polarized (CP) light, their interactions are very different [156]. Regardless of the prevalence of chirality in nature, chiroptical effects are typically relatively feeble because of the substantial disparity between the molecule size and incident optical wavelength. Circular dichroism (CD) is a strong differential in the absorption of light with distinct circular polarizations that is produced by chiroptical metasurfaces [157,158]. The chirality parameter, κ, can be used to fundamentally explain the propagation of light in a reciprocal isotropic chiral medium [159,160]:

(4) $$\left(\begin{array}{c} D/\varepsilon_{0}\\ cB \end{array}\right)=\left(\begin{array}{cc} \varepsilon_{r} & i\kappa \\ -i\kappa & \mu_{r} \end{array}\right)\left(\begin{array}{c} E\\ \eta_{0}H \end{array}\right),$$

where *D* is the field of electric displacement, *E* is the electrical field, *H* is the magnetic field, *B* is the magnetic induction field, *c* is the speed of light, $\varepsilon_{0}$ is the vacuum permittivity, $\varepsilon_{r}$ is the relative permittivity, $\mu_{r}$ is the relative permeability, and $\eta_{0}={\left(\mu_{0}/\varepsilon_{0}\right)}^{1/2}$ is the impedance of a vacuum wave. For the above equation, the pseudoscalar κ, a genuine scalar-like variable that experiences a sign conversion under parity inversion, correlates with the true vectors and pseudovectors. This medium has the following RI [160,161]:

(5) $$n_{\pm }={\left(\varepsilon_{r}\mu_{r}\right)}^{1/2}\pm \kappa ,$$

where “+” and “−” represent the right CP (RCP) and left CP (LCP). Thus, it is feasible to convey typical chiroptical signals, such as optical rotatory dispersion (ORD), the rotation of the polarization direction, and CD, owing to the differential absorption of CP with the RI [160,161]:

(6) $$ORD=\frac{2\pi l}{\lambda }Re\left(n_{+}-n_{-}\right)=\frac{2\pi l}{\lambda }Re(\kappa ),$$

(7) $$CD=\frac{2\pi l}{\lambda }Im\left(n_{+}-n_{-}\right)=\frac{2\pi l}{\lambda }Im(\kappa ),$$

where *l* is the medium’s length and λ is the wavelength.

**Figure 11:**
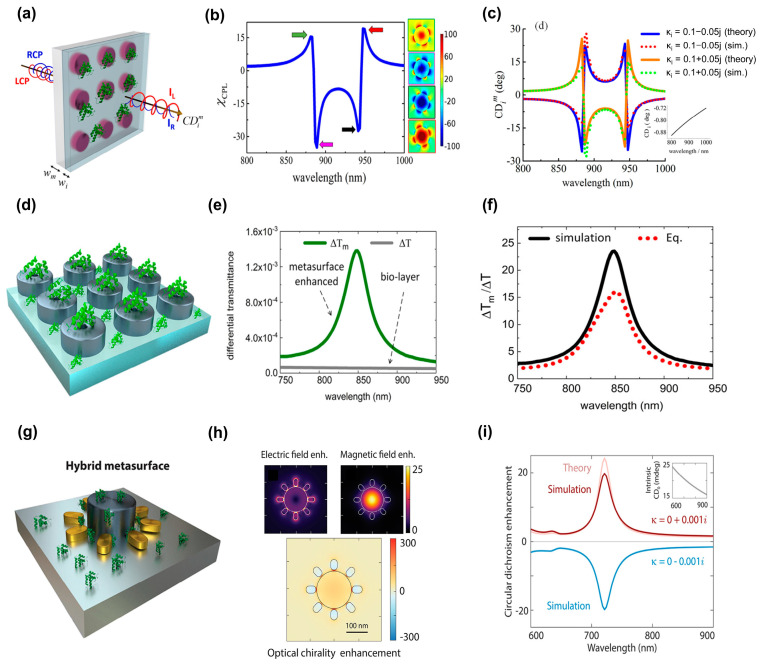
Chiral sensing. (**a**) Chiral sample atop the desired substrate. (**b**) Nanophotonic substrate’s average optical chirality enhancement factor and the local optical chirality enhancement at the ED and MD resonances, directly above the nanodisk (displayed in color on the panels). (**c**) CD signal computed for two chiral samples using simulations and analytical theories. (**a**–**c**) Reproduced with permission from [167]. Copyright © 2018 American Chemical Society. (**d**) All−dielectric chiral sensor comprising 300 nm diameter holey Si disks organized in periodic fashion with a 500 nm lattice constant. (**e**) Differential transmittance of the chiral sensor (green) with a single 150 nm chiral biolayer (gray). (**f**) Numerical calculation of chiral signal enhancement in differential transmittance. The eq. is $\frac{∆T^{m}}{∆T}=\frac{C_{sample}^{av}}{C_{RCP}}$ where $C_{sample}^{av}$ is the averaged optical chirality and $C_{RCP}$ is the optical chirality of the incident RCP light. (**d**–**f**) Reproduced with permission from [169]. Copyright © 2019 American Chemical Society. (**g**) For improved chiral molecular detection, unit cell with a Si disk encircled by gold nanorods. (**h**) Enhancements in the electric, magnetic, and optical chirality at resonance (λ = 720 nm) on a plane in the middle of the nanorods. (**i**) Increased circular dichroism signals from a metal and hybrid dielectric metasurface. (**g**–**i**) Reproduced with permission from [171]. Copyright © 2021 The Authors. Published by American Chemical Society.

As activated by various CP light sources, chiral molecules exhibit varied optical characteristics and have a non-superimposable mirror image. CD is used to identify chiral molecules, which provides crucial physical and chemical properties in protein function and cell communication. Molecular chiral optics enable the label-free, non-invasive, and inexpensive identification of chiral compounds by spectroscopic measurements [162,163]. The CD approach, which relies on the difference in how opposite enantiomers absorb left- and right-CP light (L-/R-CPL), is a popular technique for identifying chiral molecules [164]. The standard CD enantiomer spectroscopic methods, on the other hand, appear to have limited sensitivity perception of the weak chiroptical signal owing to the scale mismatch between the helical pitch of chiral molecules and the optical wavelength. This means that to reliably detect the weak chiral signals of molecules, high-concentration solutions, powerful lasers, or high-precision analytical tools are needed. By producing superchiral fields with significant optical chirality, C [165], metasurfaces can enhance the light–matter interaction in chiral sensing, and C can be defined as:

(8) $$C\left\{E,H\right\}=\frac{-k_{0}}{2c_{0}}Im\left\{E.H^{*}\right\},$$

where $k_{0}$ and $c_{0}$ represent the wavenumber and speed of light in a free space, whereas E and H are the complex electric and magnetic field vectors, respectively [166]. To clarify the crucial design parameters and reveal the associated trade-offs, Mohammadi et al. developed an analytical model to describe the interaction between the properties and performance of any given metasurface. The analytical model stipulates the following standards for the nanophotonic platform’s optimal chirality detection performance: (a) Eliminating the background signal from the detecting equipment while accounting for the chiral absorption’s function in attenuation, (b) Equivalent intensification factors for the R- and L-CPL to eliminate permittivity dependence in the overall output signal, and (c) High optical chirality to increase the output signal. The enhancement of the detection sensitivity of chiral molecules is restricted by interference from the baseline CD spectrum and the spectral drift of the chiral metasurface caused by molecules being introduced into the near-field of the plasmonic structure. Therefore, to prevent disturbance by the chiral response of the nanostructure itself, the achiral metasurface is devised. An all-dielectric chiral metasurface constituting a Si nanodisk (Figure 11a) was developed by Mohammadi et al. [167] for CD signal augmentation and extraction analysis. The chirality-enhancement factor for the proposed platform was investigated (Figure 11b). It is clear that the ED and MD resonances can produce cylindrical resonator significant optical chirality within the two resonances as well as substantial optical chirality at their respective wavelengths. At multipolar Mie resonances, these chiral near-fields are visible in dielectric materials with high RIs [168]. Because plasmonic chiral structures typically have great optical chirality, this dielectric achiral substrate nevertheless delivers that. The sign of the optical chirality is flipped at the two sides of both resonances, which indicates that the sign of the CD signal will also shift once a biolayer is placed on top of the substrate. Additionally, the authors also determined the CD signal theoretically (solid blue) and through full-wave simulations (dotted red) (Figure 11c). It is evident that there is great agreement between the theoretical and simulation results. The CD signal of the biolayer without a substrate is approximately 0.80°, as illustrated in the inset of Figure 11c. It should be emphasized that this platform offers extraordinarily high CD enhancement, of the order of 30 at the resonances and of 10–20 between the resonances, in addition to eliminating the effects of the background noise and dielectric constant.

Furthermore, the detection or discrimination of low-abundance enantiomers is of immense importance in the biological field. However, the molecular chirality is much lower than the linear RI, resulting in low detection efficiency. Recent studies have shown that chiroptic signals can be enhanced by increasing the optical chirality of the electromagnetic field and the interaction with chiral samples. Based on this, an optimal chiral sensing platform was developed by creating a highly chiral near-field with a uniform dielectric structure [169]. Spectrally and spatially overlapping electric and magnetic resonances with a π/2 phase-difference are tailored along with the Kerker effect and the duality concept to maximize the C improvement. A dielectric metasurface composed of periodic (lattice constant, 500 nm) holey disks is constructed after realizing Si disks’ capacity to provide powerful chiral fields. The chiral biolayer covering the resonators and the glass substrate is assumed to be attached to this metasurface for chiral-sensing applications (Figure 11d). When the system is sequentially illuminated with R- and L-CPL, the enhanced differential transmission (T^m^) of the chiral sample on top of the metasurface for h = 130 nm can be obtained and calculated (Figure 11e). The promise of this method for ultrasensitive nanophotonics-based chiral sensing is underlined by the ability of such dielectric metasurfaces to increase the T^m^ signal of a thin chiral analyte layer by more than one order of magnitude. Through analytical calculations, the degree of enhancement is determined, revealing a striking correspondence with the outcomes of comprehensive full-wave simulations (Figure 11f).

In biology, enantiomer discrimination and detection are essential for drug development and clinical pharmacology [165,170]. However, because chiroptical signals are inherently weak, chiral sensing has major limits. Metaphotonics is a possible approach for boosting sensitivity due to the increased optical chirality amplified by strong electric and magnetic fields. Mohammadi et al. suggested combining them in a synergistic way to create hybrid metal–dielectric nanostructures that will take advantage of their dual nature for superchiral fields for ultrasensitive chiral detection [171]. Many nanorods organized as a hybrid oligomer have been employed to improve the overall volume of the chiral hotspots. For example, eight identical gold nanorods are evenly spaced, encircling a core Si disk (Figure 11g). At the disk’s magnetic resonance wavelength, powerful electric hotspots are produced by the associated electric resonances in the nanorods and the disk (Figure 11h, top). Moreover, These spectrally overlapping resonances display electric and magnetic fields that are dephased by π/2, resulting in the optical chirality of every nanogap by 300 times (Figure 11h, bottom). The hybrid metasurface boosts CD 20 times more than a chiral layer devoid of nanostructures, because hotspots with intense local optical chirality occur, and the CD enhancement is calculated in a way that agrees well with numerical simulations (Figure 11i, pink and red). The CD also reverses when the handedness of the chiral molecules is reversed, proving that the structure does not make any chiroptical background. The potential for strong fields in metal–dielectric nanogap antennas will drive the sensitivity of chiral molecular detection for small molecular quantities.

In summary, we have explained the capacity of SPR-based platforms for high-sensitivity and diagnostic applications. Nevertheless, it is noteworthy that these platforms confront hurdles in the realm of high-throughput assays and the miniaturization of sensors. Conversely, nanophotonic approaches based on LSPR exhibit exceptional performance in multiplexed and parallel assays while also facilitating seamless integration into POC devices and clinical diagnostics on chip. Nevertheless, they frequently demonstrate lower sensitivities in comparison to techniques such as SEF and vibrational spectroscopy. Vibrational spectroscopies—specifically, infrared absorption and Raman scattering methods—can serve as valuable complements to refractometric biosensors, which offer selectivity without the need for analyte-specific receptors and also provide valuable information regarding the molecular structure of the analyte. These techniques stimulate molecular vibrations, the resonant frequencies of which are contingent upon the chemical composition of the bonds and their conformations. Hence, they can be utilized to detect molecular fingerprints and scrutinize molecular conformations in the absence of external labels. The major disadvantage in implementing vibrational spectroscopies for small-volume biological samples lies in their relatively limited sensitivity. Metaphotonic structures have the capability to enhance analyte signals through surface-enhancement mechanisms, employing techniques such as SEIRA and SERS. Recently developed all-dielectric metaphotonic platforms propose a solution to the limitations of low-resonance Q factors and heat generation. They hold the potential to provide higher sensitivities and improved performance in heat-sensitive biological systems. However, there is still a considerable scope for enhancing the performance of these dielectric structures in practical biosensing applications. One potential avenue for improvement is the customization of the spatial near-field overlap with bonded molecules. Another crucial consideration is the discrimination of enantiomers, which possess identical molecular formulas but exhibit mirror-image molecular structures. However, for the majority of natural molecules, circular dichroism signals are typically quite small, resulting in inherently weak chiral signals. To address this, the field enhancement capabilities of metaphotonic structures can be harnessed for the development of chiral sensing with remarkable sensitivity.

## 4. Chip Integration in Metaphotonic-Based Biosensing

### 4.1. Optofluidic Metasurface Biosensors

Unique possibilities for regulating flows of analytes in micro- and nanoscale volumes are now possible thanks to advancements in fluidics and nanotechnology. The ability to engineer such fluidic effects has become crucial in maximizing the surface binding efficiency for analyte systems with extremely low concentrations. Research efforts for POC systems have primarily focused on developing compact metaphotonic devices with a small footprint at the nanoscale level. Understanding the background and mechanics of fluidic behavior and manipulation in extremely small volumes is crucial for biosensing applications, particularly when combining fluidics with metaphotonic signal transducers.

**Figure 12:**
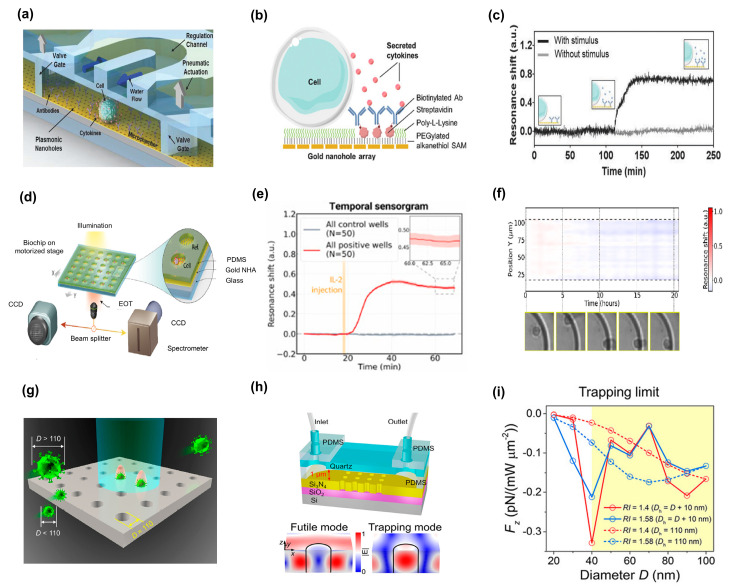
(**a**) Optofluidic sensor integration for detecting the NS1 protein directly from blood. (**b**) Approach to surface functionalization for cell binding and in situ cytokine detection. (**c**) Real-time sensorgrams from a single EL4 cell that secretes IL-2 cytokine in response to chemical stimulation (black) and a negative control without the stimulus (gray). (**a**–**c**) Reproduced with permission from [174]. © 2018 WILEY-VCH. (**d**) Integrated biochip and measuring equipment for obtaining a spectroscopic and bright-field optical microscope. (**e**) Temporal sensorgram of the complete microarray averaging across 50 positive wells and 50 control wells. (**f**) Spatiotemporal sensorgram of IL2 secretion for one EL4 cell and magnified images of one EL4 cell in the control group in the microwell at different time points. (**d**–**f**) Reproduced with permission from [60]. © 2022 Elsevier. (**g**) Viral manipulation using arrays of resonator nanocavities. (**h**) Optofluidic viral chip with a shallow microchannel of 1 µm and the electrical fields of the Trapping mode, which traps light inside a hole, and the Futile mode, which traps light inside a solid Si_3_N_4_. (**i**) Trapping limit on viruses with a diameter of 20 to 100 nm along with the RI of the virus (1.4) and polystyrene nanoparticle (1.58). (**g**–**i**) Reproduced with permission from [61]. © 2022 Wiley-VCH.

One of the most effective methods for creating novel treatments for critical diseases, including cancer or autoimmune disorders, is single-cell analysis [172]. Label-free biosensors offer a novel approach to the sensitive, precise, and in-the-moment measurement of cell secretion [66,173]. This detection method avoids the time-consuming and expensive molecular tagging and enables the non-intrusive and dynamic elucidation of biomolecular interactions. The creation of highly improved optical biosensors based on metaphotonic nanostructures has been sparked by recent advancements in nanotechnology. Metaphotonics enables extremely low detection limits, extraordinary downsizing, and the best on-chip integration by facilitating strong light–matter interactions and substantially restricting light in small volumes. By investigating the dynamic secretion mechanism at the single-cell level, it is possible to reveal the real-time functional status of specific cells. The laborious molecular labeling required by fluorescent and colorimetric methods limits the temporal resolution and inevitably interferes with cell integrity. A label-free nanoplasmonic biosensor was presented as a novel method for the real-time analysis of single-cell secretion [174]. The nanobiosensor uses an innovative design for a microfluidic system based on a gold nanohole array with a small-volume microchamber and regulatory channels for the hours-long accurate tracking of cytokine production from individual cells (Figure 12a). The entire surface of the sensor is uniformly covered by nanohole arrays using low-cost, wafer-scale photolithography. The regulated flow concurrently reduces evaporation, stops bubble forming, and enables a stable optical background signal to facilitate the dependable monitoring of single-cell secretion analysis. The functioning of the biosensor relies on the extraordinary optical transmission (EOT) phenomenon, where regularly spaced subwavelength nanohole structures significantly enhance light transmission [175,176]. The identification of light amplification is indicated by the appearance of a resonance peak in the transmission spectrum due to EOT. The near-field RI across the surface of the nanohole governs the EOT resonance. The sensor module’s surface passivation and functionalization are equally important for increasing the specific analyte binding efficiency. Typically, non-specific protein adsorption on the sensor surface makes the detection of minimally treated raw biological materials difficult. To address this, an antifouling self-assembled monolayer (SAM) was used to functionalize the optical sensor surface prior to the immobilization of probe molecules for capturing cells and targets (Figure 12b). There is a clear resonance shift in response to an external chemical stimulation, demonstrating that the optofluidic sensor can quickly identify the protein release from individual cells (Figure 12c).

Even though nanoplasmonic sensors, as demonstrated by Li et al. [174], exhibit the capability to detect secretion at a single-cell level, the measurements on individual cells need to be performed one at a time. To conduct real-time investigations of the secretomes of multiple cells with a single-cell resolution, it is imperative to develop new tools that can reveal the individual cellular activities and the underlying kinetics. Liu et al. [60] aimed to achieve the label-free and real-time monitoring of secretome on a large scale using a high-throughput and ultrasensitive nanoplasmonic biosensor (Figure 12d). The gold nanohole array based on the nanoplasmonic substrate displayed an extremely sensitive spectral response to localized variations in RI resulting from the binding of analytes to its surface. The authors carried out a binding test on a microarray with 10 × 10 microwells (100 μm in diameter) to illustrate the high-throughput dual imaging approach and the creation of time-resolved two-dimensional resonant shift information. The temporal sensorgram of the whole microarray is displayed in Figure 12e, averaging 50 positive wells and 50 control wells, with the shaded region for each curve representing three times the standard deviation. The functionalized biochip responds to the presence of analytes in a uniform manner, as seen by the low variance. This microarray binding assay highlights the high throughput of the platform to scan a wide region and analyze numerous wells simultaneously. The cells were exposed to both a mixture of stimulants and a cocktail of protein transport inhibitors during the control experiment. The outcome of one cell from this group is displayed in Figure 12f (top). Throughout the testing, the spatiotemporal sensorgram exhibited light color, implying a low level of interleukin-2 (IL-2) release. The cell is feasible, as shown in Figure 12f (below), and displays a little bit more displacement. Because there was no resonance response in the negative control group, it is concluded that their biosensing platform only detects IL-2 and does not react to any other proteins in the culture environment. Moreover, this device is perfect for basic cell research and clinical studies with promising applications in diagnosis, tailored cell therapy, and drug screening.

In-depth studies of virus pathogenesis and evolution require the precise manipulation of a specific quantity of viruses to infect host cells spatially. Despite advancements in optical tweezers that enable optical cooling to the atomic level, the effective trapping and manipulation of viruses in an aqueous environment are still challenging owing to weak optical forces and a lack of multifunctional spatial manipulation techniques. Shi et al. [61] have demonstrated the effective manipulation of both small (approximately 40 nm) and large viruses using nanocavity arrays facilitating the trapping mode. A significant step toward large-area viral manipulation and greater optical forces compared to those in typical photonic crystals (based on the Bloch mode instead of defect cavities) has been realized due to the balance between subwavelength confinement and light leakage. A large-sized Gaussian beam (532 nm) is projected onto a 110 nm diameter array of Si_3_N_4_ nanocavities to stimulate the trapping mode in the dielectric nanocavities (Figure 12g). The term “Trapping mode” alludes to the behavior of a virus particle as it approaches the illuminated area and gets trapped in the beam. Smaller viruses are contained inside the nanoholes, whereas larger viruses are discharged when the laser turns off due to the microfluidic drag force. An optofluidic multifunctional virus chip with shallow 1 μm microchannels was fabricated to ensure that viruses flowing into the laser-illuminated area will have sufficient optical force to pull them to the surface and nanoholes (Figure 12h, top). The nanocavities are made to form the trapping mode, which is used to trap viruses confined inside it when the intense light is focused to provide a powerful optical gradient force (Figure 12h, bottom). On the other side, the “Futile mode” develops when light is directed along the dielectric layer rather than getting trapped inside the nanohole, resulting in a far weaker optical gradient force that is unable to trap viruses. Additionally, the trapping force on viruses (diameters varying from 20 to 100 nm) and viral RI (1.4) and polystyrene nanoparticle RI (1.58) was examined to determine the trapping limit of the nanocavities with D (the diameter of the nanoparticle) and D_h_ (nanohole diameter) (Figure 12i). For D < 40 nm, the trapping force is insignificant; however, it is noticeable for D ≥ 40 nm. This indicates that 40 nm is the trapping limit of the nanocavities in the Trapping mode. Precision management of the microenvironment conditions during host–cell infection and drug testing, when combined with the established microfluidics and lab-on-a-chip technologies, may increase the effectiveness of vaccination and antiviral drug research.

### 4.2. High-Q Metasurface Biosensors

The benefits of metasurfaces that enable high-Q resonances include strong electromagnetic fields, spectrum selectivity, and better light–matter interactions for fluorescent spectroscopy [177]. They enable differentiating chiral molecules [178]. Further, numerous mechanisms, such as lattice [179] and Fano resonances [180,181], as well as BIC [55,80,182,183], can be adopted for metasurface biosensing.

**Figure 13:**
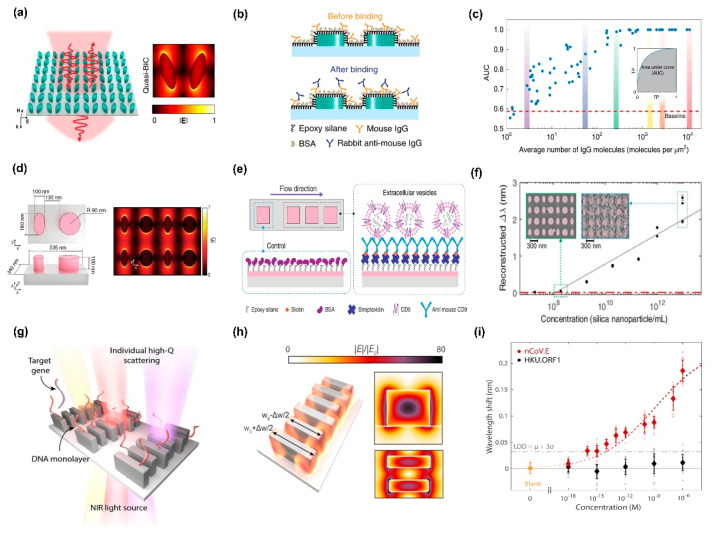
(**a**) Silicon elliptic zigzag arrays with high-Q BIC-induced resonances and quasi-BIC mode distribution in the electric field. (**b**) Representation of the bioassay for biomolecule immobilization using epoxy-silane-based covalent chemistry. (**c**) Biomolecules detection utilizing image-based data processing with dielectric metasurface sensors. (**a**–**c**) Reproduced with permission from [184]. Copyright © 2019, The Author(s). Springer Nature. (**d**) View of the metaunit produced by a cylindrical and an elliptical a-Si resonator and distributions of the electric field in four adjacent meta units from numerical calculation. (**e**) Extracellular vesicles detection using a biorecognition assay on the metasurface sensors. (**f**) Reconstructed spectral shift calibration of 100 nm diameter biotinylated silica nanoparticles. (**d**–**f**) Reproduced under CC BY Creative Commons Attribution 4.0 International License (https://creativecommons.org/licenses/by/4.0 accessed on 20 April 2023.) [58]. Copyright © 2021, The Author(s). Springer Nature. (**g**) Metasurface arrays of a high-Q factor containing Si blocks interfaced with DNA probes to detect specific genes. (**h**) Electric near-field augmentations in simulation for a resonator with ∆w = 50 nm. (**i**) Concentration-dependent binding reactions for the SARS-CoV-2 envelope (nCoV.E) and the open reading frame 1b (ORF1) targets incubated on metasurface devices. (**g**–**i**) Reproduced under CC BY Creative Commons Attribution 4.0 International License (https://creativecommons.org/licenses/by/4.0 accessed on 20 April 2023.) [59]. Copyright 2021, arXiv.

In terms of BIC in dielectric metasurfaces [80], the unit cell in-plane symmetry breaking, using a manipulated asymmetric parameter, can contribute towards quasi-BIC modes with a very high Q factor. Lately, a BIC dielectric metasurface has been employed in hyperspectral imaging and data analysis techniques to optimally process spatially resolved spectra from millions of image pixels for ultrasensitive biomolecule detection [184]. Their light scattering-based resonant metasurface entirely composed of pairs of tilted silicon nanobars (Figure 13a, left) was manufactured via a complementary metal-oxide semiconductor (CMOS)-compatible processes. In the design, the authors make the most of the quasi-BIC states, which are gained with small but finite values of the tilting angle (17.5°), inducing high-Q and spectrally isolated resonances with high suppression. The resultant robust light trapping happens in such a way that electric fields in the metaunit exhibiting the quasi BIC mode are localized in the outer volume around the nanostructure (Figure 13a, right), making it ideal for sensing applications. To investigate the affinity with all-dielectric metasurfaces for biomolecular realization assays, epoxy-silane chemistry was used to covalently immobilize the catch molecules onto the sensor surface. The biosensors were incubated with rabbit-originated anti-mouse IgG (R-IgG), which has a great affinity for M-IgG, to ensure the binding of surface-immobilized M-IgG molecules to R-IgG (Figure 13b). Such prototypical bioassay makes a concession of the quantification of the molecular density and the determination of metasurface detection limits. Both are independent of bioassay affinity factors. The metasurface was optimized to provide resonance at approximately 850 nm; it displayed a Q factor as high as 144 reachable and robust near-fields and demonstrated an S of ~263 nm RIU^−1^ and an FOM of ~40 RIU^−1^. To evaluate the high-performance sensing, a threshold approach, the Receiver Operating Characteristic (ROC) curve, was conducted. The result (Figure 13c) manifested the detection of IgG molecules as low as three molecules per μm^2^, which corroborates the advantage of the label-free imaging technique at the diffraction limit and multiplexed biorecognition in a single measurement.

Recent research has focused a lot on quasi-BIC. By using quasi-BIC modes, it is possible to tailor the resonance bandwidth, spectral positions, Q factor, and distribution of electric and magnetic fields among the meta-atoms with a considerable deal of flexibility [79]. For example, meta-atoms that constrict the fields inside the resonator volume can achieve Q factors of up to tens of thousands and are appealing for creating nanoscale light sources [185,186] and beam steering with spectral selection [13]. Metasurfaces with Q factors of a few hundred are attractive for sensing applications [38,154] because they offer substantial field improvements outside the resonator. Spectrometers and wavelength-scanning devices offer reliable spectral shift data for tracking changes in the optical response; however, they are cumbersome and pricey. As a result, they may not be used to collect time-resolved images for calculating molecule binding kinetics. Additionally, spectrometers are restricted in their ability to execute multiplexed bioassays by simultaneously gathering data from a variety of sensors. Thus, straightforward and scalable optical read-out systems, which retrieve both time and spatially resolved information from vast sensor regions, are necessary. A spectrometer-free single-wavelength imaging biosensor based on dielectric metasurfaces was developed by Jahani et al. [58] to achieve exceptional sensitivity relying on a reconstructed spectral shift. To realize this, a diatomic dielectric metasurface is needed that offers a quasi-BIC mode and breaks in-plane symmetry to enable high-quality resonance with reachable near-fields [79]. A dimer-type unit cell “meta-molecule” serves as the foundation for diatomic metasurfaces. The dimer symmetry is broken by altering the ellipticity of one of the meta-atoms to facilitate high Q resonance and robust field-analyte overlaps. Such diatomic structures offer superior engineering flexibility for the in-plane asymmetry as opposed to single-unit metasurfaces while maintaining a simple design and applicability for biosensing applications. The meta-molecule has an elliptic and circular disk, which enables breaking the in-plane inversion symmetry (Figure 13d, left). The degree of ellipticity of one of the meta-atoms, which is inversely proportional to the difference between the ellipse long and short axes, describes the asymmetry. Their construction provides readily accessible augmented electric fields in the exterior surface of the dielectric nanoresonator (Figure 13d, right). For the in-flow and real-time detection of breast cancer extracellular vesicles containing exosomes, the large-area antibody functionalized metasurface chip was set up as a microarray and coupled with microfluidics on the imaging platform (Figure 13e). This biosensor enabled the detection of approximately 0.41 nanoparticles/µm^2^ and the observation of extracellular vesicle binding in real time at concentrations as low as 204 femtomolar solutions, which is equal to 1.23 × 10^8^ particles/mL (Figure 13f). Such a technique creates new fertile ground for reliable and ultra-sensitive POC devices, which can provide temporally and spatially resolved spectral shift information by combining the ease of intensity-based large-area and real-time image detection with robust spectroscopic systems.

Significant improvements in the diagnosis, monitoring, and treatment of organism and ecosystem health have been realized through genetic screening techniques. For instance, the health of waterways, cattle, soil, and air are monitored by new environmental DNA sensors [187], whereas tissue and liquid biopsies identify malignant genetic alterations and the possibility of recurrence and are used to define treatment [188]. Biosensors built on nanotechnology hold the potential of novel platforms for rapid and scalable biomolecule detection without the need for target labeling or biochemical amplification. However, the use of fluorescence and absorbance in current nucleic acid technologies, such as PCR and DNA microarrays, requires sample amplification or replication, increasing the processing time and cost. To surpass these disadvantages, a platform for label-free genetic screening based on high-Q Si nanoantennas functionalized with monolayers of nucleic acid fragments was investigated (Figure 13g) [59]. The extensive biosensor integration resulted from a significant electromagnetic field increase in each nanoantenna, with sufficient localization ensuring separation from nearby resonators. Remarkably, electric fields are 80 times stronger at the surface of Si blocks (Figure 13h). By combining the resonators with desired probe DNA sequences, they could identify target genes with more specificity. Experimental resonant shifts ranged from 0.2 nm at 1 µM to 0.01 nm at 1 aM (Figure 13i). The LOD of approximately 8 fM recorded from this device marks a significant improvement compared to earlier work on metaphotonic nucleic acid sensors [189,190,191]. This sensor offers promise for amplification and label-free viral diagnostics with a detection limit in the low femtomolar domain.

To conclude this section and provide a more comprehensive outlook, a comparison of chosen contemporary instances of metasurface biosensors based on metaphotonics, categorized by the configuration, target analyte, and detection limit, is tabulated in Table 1.

## 5. Challenges and Potential Directions

### 5.1. Cost-Efficiency

To prevent problems of cross-contamination and challenging cleaning processes while handling biological materials, affordable disposable biosensor chips would be ideal. For example, in low-resource circumstances, Tao et al. [201] presented an inexpensive, disposable, and simple-to-use split ring resonator sensor on a paper substrate for the quantitative determination of blood glucose. Recently, Leitis et al. [202] developed a microfluidic system for real-time label-free biosensing using aluminum-based plasmonic metasurfaces, which might be suitable for disposable medical devices. Because of smartphones’ capabilities, there is a tendency to combine nanophotonic biosensors with them to lower prices and ease wide-scale dissemination [203,204]. These sensors can be employed to analyze information derived from patients’ samples through customized apps, quantify the signals, and then wirelessly transmit the results to medical professionals for analysis. Even though planar-integrated biosensors could potentially possess a smaller footprint and portability, they will be costly in single-use scenarios owing to the intricate manufacturing process required for generating multiple device layers and packaging phases. Because diverse designed nanostructures can be patterned in a variety of ways, researchers have mainly used electron-beam or focused-ion-beam lithography in developing metaphotonic biochips. Nevertheless, the current serial patterning technologies have a low throughput and excessive costs, which necessitates the need for low-cost alternative manufacturing techniques to enable commercialization. Utilizing Moore’s law, which involves using infrastructure and manufacturing practices compatible with Si, is a potential approach. Materials present a significant problem in this regard; for instance, front-end CMOS processing is incompatible with gold and silver. Inexpensive and large-scale top-down lithography techniques, such as nanoimprinting and deep and extreme UV lithography, will receive greater emphasis in alternative manufacturing strategies [205,206,207,208].

### 5.2. Sample Processing

Because microfluidic devices enable activities, such as sample handling, sample concentration, and analyte transport, in addition to reducing the necessary specimen volume and the use of pricey chemicals, they are advantageous for biosensor integration [209]. Prolonged detection periods may happen because limited mass transport causes analytes to move slowly from bulk samples to hot spots. Metaphotonic biosensors exhibit high sensitivity through field confinement in subwavelength hot spots. Currently, some methods have been proposed to solve this matter, including the integration of external lasers, electrical fields, and microfluidic modules to facilitate the analyte concentration and transport to the sensor surface [210,211]. Another challenge is the on-chip integration or removal of costly and bulky external microfluidic components, such as valves and pumps. The promising alternative is capillary microfluidics, which may deliver liquid samples, without an additional equipment requirement, by leveraging surface-tension effects [212]. Digital microfluidics is a new technique that manipulates microdroplets employing electric forces [213]. Crucial factors in on-site biosensing are sample collection and processing. The diverse range of analytes and matrix composition, such as biological fluids in medical diagnostics and food samples in food safety, present significant difficulties. Biomarker detection commonly involves the use of blood as the primary biofluid. Though blood collection is minimally intrusive, it remains necessary to isolate blood components and mitigate the impact of interferences on matrix effects [214]. The usage of biofluids for bioanalysis, which contain simpler chemicals than blood and can be collected without causing harm (for example, saliva, perspiration, or urine), is becoming increasingly popular [215].

### 5.3. Chip Miniaturization

Metaphotonic biosensors that offer much better downsizing, portability, and throughput are being developed because of advancements in integrated photonic circuits. The mobility and resilience of the sensor are significantly improved by the full monolithic integration of all sensor elements, including light sources, spectrometers, and fluidic motors. This is particularly important for field-deployed applications in areas with scarce resources. Recent studies employing intensity, phase, lens-free, or hyperspectral imaging techniques indicate that stimulating resonant nanostructures with typically incident light along a collinear optical channel can be advantageous for miniaturization and multiplexing [204,216]. Metasurface-based sensing technologies can address the downsizing of current bioanalytical and clinical equipment. Timely diagnosis is crucial for the prompt, efficient, and accurate treatment of life-threatening illnesses. POC testing devices and ongoing health monitoring played a key role during the COVID-19 pandemic for preserving life and enhancing quality of life [217,218]. CMOS-based chips [32,219] and microfluidic devices [149,195] have been integrated with plasmonic metasurfaces. Dielectric metasurface integration is still in its initial stages; however, it is anticipated to take advantage of collected expertise to enable additional functions. For example, pixelated [55] and reconfigurable metasurfaces [220,221] can facilitate tiny sensors via the integration of on-chip light generation, signal processing, and surface augmentation.

### 5.4. Specificity

The optical sensing is substantially simplified because numerous commonly used optical biosensors work in homogenous media, such as water or a buffer solution. Because of the strong background signals by the biological matrix, the direct detection of biomarkers in bodily fluids without sample pre-processing presents substantial hurdles when sensing in complex heterogeneous media. To solve the problem, surface chemistry and the desired optical sensing materials must be used to execute the surface functionalization of the optical sensors. Hot spots—regions of intense electromagnetic fields—are created by nanophotonic structures because their surfaces enable tight light confinement within dimensions smaller than ten nanometers. The nanophotonic affinity biosensors often need the lowest thickness (10–20 nm) of antifouling coating for the desired operation, which can keep immobilized receptors outside hot spots. Although high-affinity receptors, such as aptamers [222] and nanobodies [223], are advantageous for stronger interactions, molecular switches exhibiting reversible interactions have been proposed for continuous biosensing [224,225]. Additionally, metasurface-based Plasmonic Resonance Energy Transfer (PRET) hyperspectral imaging is used to explore biological light–matter interactions [226].

### 5.5. Advancing Metaphotonic Biosensors Assisted by Artificial Intelligence

Recently, there has been a significant surge in research at the intersection of photonics, machine learning (ML), and artificial intelligence (AI) [227]. A novel methodology can be employed to characterize various photonic systems, such as optical waveguides, nanoantennas, and metasurfaces. These groundbreaking approaches establish the core principles of the interaction between light and matter, facilitating the intelligent design of metaphotonic sensing devices. Metasurface-based biosensing, as a highly promising and rapidly advancing field in modern photonics, has incorporated the use of AI-assisted techniques [145]. In this scenario, ML can be utilized to optimize sensors by employing metasurfaces [228] and to achieve accurate analytical results from noisy and low-resolution sensing data that may exhibit significant overlap [229]. Furthermore, ML plays a crucial role in enabling direct, automated, accurate, and rapid biosensor readout, which is of great significance for on-site detection or diagnosis. To demonstrate the application of ML in biomolecule classification, we highlight the recently proposed platform for SEIRA that incorporates deep learning (DL) algorithms [40]. They utilize a nanoplasmonic metasurface with three resonances spanning a wide mid-IR spectrum (1000–3000 cm^−1^), which encompasses the absorption bands of biomolecules. By utilizing real-time measurements of reflectance spectra, absorbance can be calculated and subsequently analyzed using a DL technique. This allows the algorithm to differentiate between each analyte at every time point. Metasensors have been employed to tackle global challenges, including the COVID-19 pandemic [230]. AI technologies have been applied to diagnose the coronavirus [231], and there are proposals to integrate them with meta-sensors. Notably, the SARS-CoV-2 saliva sensor showed outstanding sensitivity and specificity, both achieving 95.2%, in clinical trials [232]. Developing metasurfaces with desired properties and functions relies on innovative design strategies and state-of-the-art computational techniques. Therefore, ML has the potential to revolutionize metaphotonics devices by enabling the discovery of unique optical designs, leading to advancements in sensing and other functionalities in the near future.

## 6. Concluding Remarks

In this review, we provide an overview of the essential principles, distinctive characteristics, and influential factors associated with several significant resonance modes generated by the interaction between electromagnetic waves and plasmons or dielectric meta-atoms. Metaphotonic biosensors offer the ability to generate amplified near-fields in nanoscale volumes around the sensor surface, enabling remarkable sensitivity to changes in the local environment due to molecular binding and adsorption. The metaphotonic structure, engineered to operate across a wide electromagnetic spectrum, can be designed to facilitate refractometric sensing, SERS, and SEIRA. Chiral sensing with metasurfaces can generate prominent electric and magnetic dipole moments. These metasurfaces have recently gained significant attention, as they offer the potential to produce superchiral near-fields, which can be utilized for the detection and differentiation of chiral biomolecules. The effective utilization of nanophotonic hotspots necessitates the precise customization of the analyte flow, highlighting the criticality of employing microfluidic approaches. Thus, we have discussed the essential facets of optofluidic engineering based on plasmonic metasurfaces and BIC-mode dielectric metasurfaces, encompassing multiplexed high-throughput assays and expedited analyte-specific binding to achieve lower detection limits and enabling single-molecule detection and rapid response times. The successful integration of metaphotonic structures with advanced functional coatings capable of selectively capturing target analytes while maintaining inertness towards other molecules is essential for ensuring the reliable analysis of complex samples and enabling operation in dynamic biological environments. Similarly, the demand for integrating metaphotonic biochips with microfluidics for sample collection and processing in bioanalytical applications will persistently increase. Finally, metaphotonic biosensing will pave the way for innovative POC clinical diagnostic device developments, which improve well-being and quality of life for humans.

## Figures and Tables

**Table 1:** Summary of biomolecular detection based on metaphotonic devices.

Sensing Method	Configuration	Target Analyte	Detection Limit	Ref.
	Si nanodisk arrays	Prostate-specific antigen	0.69 ng mL^−1^	[53]
	Si nanocylinder arrays	Prostate-specific antigen	0.83 ng mL^−1^	[100]
	Si crescent	Streptavidin	0.167 nM	[102]
	Dielectric nanohole arrays	Immunoglobulin G (IgG)	1 pg mL^−1^	[192]
Refractometry				
	Si metasurface	Extracellular vesicles	133 × 10^−15^ M	[58]
	Gold nanohole arrays	Cytokine secretion	IL-2: 39 pg mL^−1^	[174]
	Si elliptic zigzag array	Anti-mouse IgG	~3 molecules/μm^2^	[173]
	Gold nanohole arrays	Exosomes	~200	[184]
	Gold nanohole arrays	Virus	<10^8^ PFU mL^−1^	[185]
	Si dimers	Single molecules	140 × 10^−21^ L	[193]
	Si metasurface	Antibody/antigen	IgG: 5 pg mL^−1^	[118]
SEF	Hybrid metasurface	IgG, cancer marker	gG: 5 pg mL^−1^	[119]
			P53: 50 pg mL^−1^	
	Si metasurface	SARS-CoV-2 RNA	100 amol mL^−1^	[194]
	Si nanodimers	β-carotenal monolayer	-	[54]
	Si_3_N_4_ cylindrical holes	R6G fluorophores	10 μM	[138]
	Plasmonic nanoslits	Nucleobase	Single-molecule	[195]
SERS				
	Gold nanopillars	N-acetylasparate	~pM	[196]
	3D nanogap pillars	Doxorubicin	10^−4^ M	[197]
	3D nanosplit rings	Hemoglobin	2.5 mg mL^−1^	[198]
SEIRA	Gold nanorods	Lipid membrane	-	[149]
	(a-Si:H) Zigzag arrays	Protein A/G	2130 molecules/μm^2^	[55]
	Ge elliptical zigzag arrays	ODAM protein	3000 molecules/μm^2^	[154]
	Metamaterial with nanogap	1-octadecanethiol monolayer	-	[152]
	Photonic crystal metasurface	Biphenyl-4-thiol	1 nm	[199]
Chirality	Si nanodisk arrays	DNA monolayers	~10^12^ molecules/cm^2^	[57]
	Metal–dielectric structures	Small molecular quantities	-	[171]
	Gold nanorods	α-Synuclein fibrils protein	80 nM	[200]

## Data Availability

Not applicable.
